# Molecular mapping and characterization of QTLs for grain quality traits in a RIL population of US rice under high nighttime temperature stress

**DOI:** 10.1038/s41598-023-31399-w

**Published:** 2023-03-25

**Authors:** Anuj Kumar, Julie Thomas, Navdeep Gill, Yheni Dwiningsih, Charles Ruiz, Adam Famoso, Andy Pereira

**Affiliations:** 1 Departemnt of Crop, Soil, & Environmental Sciences, University of Arkansas, Fayetteville, AR 72701 USA; 2 Department of Biological Sciences, Nova Southeastern University, Fort Lauderdale, FL 33314 USA; 3 H. Rouse Caffey Rice Research Station, Louisiana State University Agricultural Center, Rayne, LA 70578 USA

**Keywords:** Plant sciences, Plant stress responses, Heat

## Abstract

Elevated nighttime temperatures resulting from climate change significantly impact the rice crop worldwide. The rice (*Oryza sativa* L.) plant is highly sensitive to high nighttime temperature (HNT) during grain-filling (reproductive stage). HNT stress negatively affects grain quality traits and has a major impact on the value of the harvested rice crop. In addition, along with grain dimensions determining rice grain market classes, the grain appearance and quality traits determine the rice grain market value. During the last few years, there has been a major concern for rice growers and the rice industry over the prevalence of rice grains opacity and the reduction of grain dimensions affected by HNT stress. Hence, the improvement of heat-stress tolerance to maintain grain quality of the rice crop under HNT stress will bolster future rice value in the market. In this study, 185 *F*_12_*-*recombinant inbred lines (RILs) derived from two US rice cultivars, Cypress (HNT-tolerant) and LaGrue (HNT-sensitive) were screened for the grain quality traits grain length (GL), grain width (GW), and percent chalkiness (%chalk) under control and HNT stress conditions and evaluated to identify the genomic regions associated with the grain quality traits. In total, there were 15 QTLs identified; 6 QTLs represented under control condition explaining 3.33% to 8.27% of the phenotypic variation, with additive effects ranging from − 0.99 to 0.0267 on six chromosomes and 9 QTLs represented under HNT stress elucidating 6.39 to 51.53% of the phenotypic variation, with additive effects ranging from − 8.8 to 0.028 on nine chromosomes for GL, GW, and % chalk. These 15 QTLs were further characterized and scanned for natural genetic variation in a japonica diversity panel (JDP) to identify candidate genes for GL, GW, and %chalk. We found 6160 high impact single nucleotide polymorphisms (SNPs) characterized as such depending on their type, region, functional class, position, and proximity to the gene and/or gene features, and 149 differentially expressed genes (DEGs) in the 51 Mbp genomic region comprising of the 15 QTLs. Out of which, 11 potential candidate genes showed high impact SNP associations. Therefore, the analysis of the mapped QTLs and their genetic dissection in the US grown *Japonica* rice genotypes at genomic and transcriptomic levels provide deep insights into genetic variation beneficial to rice breeders and geneticists for understanding the mechanisms related to grain quality under heat stress in rice.

## Introduction

Rice (*Oryza sativa* L.), a major staple food, provides 35–60% of the dietary calorie intake for an estimated three billion people in the world^1–3^ and plays a vital role in food security and sustainability in Asia^4^, Americas, and Africa^5^. To meet the demands of the increasing world’s population, rice breeders and geneticists have been working on developing high yielding and improved grain quality rice varieties^6^. In recent years, rice growers and consumers have been paying attention to grain quality, along with grain yield in rice ever since climate change was recognized as a serious threat to the stability and quality of the rice crop. Based on recent statistics of Intergovernmental Panel on Climate Change (IPCC), the mean temperature of the global air surface has been increased from 1.0 to 3.7 °C in the recent years, which would potentially increase the frequency and magnitude of heat stress events^7,8^. Under these critical conditions, climate change due to global warming has increased both the nighttime temperature and daytime temperature; however, greater warming has been observed at night than during the day in rice growing regions worldwide^6,9^. High nighttime temperature (HNT) is one of the detrimental factors deteriorating grain quality year-by-year and declining the grain yield of the rice crop^6^. Several studies have shown that a rise of 1 °C in nighttime temperature reduced rice grain yield by 10% and grain quality^6^. Rice is highly sensitive to HNT stress at various phenological growth stages; however, grain filling at the reproductive stage is the most sensitive, leading to poor grain quality under greenhouse and field conditions^10–14^. In particular, HNT stress during grain filing stage increases respiration and oxidative stress in cells causing reduced flux of carbohydrates from the flag leaf to developing seeds and directly affects the grain quality^15^. A rise in nighttime temperatures has been shown to have a negative impact on grain quality and grain yield in the major rice growing areas of Asia^6^ and the United States (US)^16^. Thus, rice breeders and geneticists need to focus on grain quality characteristics in order to improve overall grain quality under HNT stress using novel genomics-assisted techniques.

Rice grain quality is a complex trait with various components, including grain appearance, milling, and eating, cooking, and nutritional qualities^17^. The appearance quality of rice grain is primarily determined by grain dimensions, a combination of grain length (GL), grain width (GW), and grain thickness (GT)^18,19^, endosperm opacity (chalkiness), chalky grain percentage, chalky area, and chalky degree (percentage chalkiness-% chalk)^20,21^. The chalky grains have opaque spots either in the endosperm ranging in size, on the dorsal side of the grain (white belly) or in the center (white corner)^22^. Moreover, several studies have reported that HNT stress affects physio-chemical attributes such as increased chalk formation^23^, reduced amylose content resulting smaller grain size in rice crop^24,25^, variation in flour protein ingredients^26^, altered the enzyme activities^11,27^, and poor starch packing resulting increased chalkiness in developing grains^28^. When rice grains are exposed to HNT stress (≥ 27 °C/80.6 °F at nighttime), 90.2% grains of *japonica* rice cultivar “Koshikihari” during 3–35 days after flowering showed milky-white and white-back^29^. The HNT stress causes impairment of starch biosynthesis and triggers inhomogeneous starch deposition resulting in loosely packed irregular and smaller starch granules in rice grains^29–32^. Recently, Shi et al.^33^ reported that HNT stress declined the head rice yield and grain width, altered the amylose content while it increased the chalkiness in several rice genotypes. Amylose content is lower under HNT stress (16.1%) compared to control nighttime temperature conditions (19.1%), suggesting that lower activity of amylose synthesis might be involved in chalk formation^29^. The grain length, grain width, and chalkiness in rice grains determine overall grain quality and price in the rice market. The genetic basis of these traits is poorly understood under heat stress conditions and the important goal lies in improving these complex correlated traits using advanced genetic tools/techniques on multi-omics datasets such as genome, transcriptome, metabolome, and proteome for genomics-assisted breeding.

Grain quality traits (GL, GW and % chalk) are polygenic quantitative traits^34^. Recent revolutionary genomic advances in QTL mapping in bi-parental recombinant inbred lines (RILs) and genome-wide association studies (GWAS) on diverse populations^35^ can resolve genetic architecture of complex traits. So far, more than 400 QTLs for grain size have been mapped across 12 chromosomes of rice, of which 109, 95, and 107 QTLs were associated with GL, GW, and 100-grain weight, respectively^36^. A few of these QTLs have been fine-mapped^37^ and a total of 14 major genes are shown to be significantly involved in controlling grain size and shape including four independent genes (*GW2, GW8, GS5, and GW5/qSW5*) for GW, eight genes (*GS2/GL2, GL3.1/qGL3, GL4, GLW7, GS3, OsLG3, OsLG3b/qLGY3*, and *TGW6*) for GL, and two pleiotropic genes (*GW6a and GL7/GW7*) controlling both GL and GW that also have larger impact on grain weight^38–40^. In last several years, many QTLs for grain appearance and eating quality have been identified in different mapping populations (RILs, DHs, CSSLs, F_2_, and BC_2_F_2_)^41^. Over the past two decades, several QTLs have been identified and are associated with grain dimension, across the chromosomes of rice^40^. More than one hundred QTLs have been related to the chalkiness trait, including 30 QTLs for percent grains with white core (PGWC), 26 QTLs for degree of endosperm chalkiness (DEC: %chalk), 12 QTLs for area of endosperm chalkiness (AEC), 11 QTLs for white-backed kernel, and 3 QTLs for basal white (BW), all distributed across12 chromosomes and are catalogued in the Gramene database^34,40,42^. A larger number of the QTLs associated with chalkiness were detected on chromosomes 5, 6, and 8 in different genetic backgrounds and environments^43^. The QTL clusters were found on the hotspot regions of these three chromosomes^44–59^. Even though HNT stress significantly deteriorates rice grain quality, a limited number of studies have been conducted to dissect the HNT stress tolerance in rice, and as a result very few QTLs/loci related to chalkiness under heat stress have been identified worldwide^60–64^. However, until now, no study has been reported on QTLs mapping for GL and GW under HNT stress in bi-parental populations in rice worldwide. Despite the increasing knowledge of QTL mapping, metabolomics^65^, and transcriptomic^66,67^ responses to HNT stress in rice, the understanding and dissection of genetic basis of grain quality traits and HNT stress tolerance in rice is still limited. Hence, based on our best knowledge and recent survey of the literature & databases, in the United States, this is the first study to identify genetic loci and putative candidate genes controlling grain quality traits and HNT stress tolerance mechanisms in *tropical japonica (TRJ)* rice under HNT stress. In this study, we investigated a recombinant inbred line (RIL) population of two elite US rice cultivars “Cypress” and “LaGrue” for grain quality traits under HNT stress. The study was carried out with the objectives: to screen and evaluate the RIL populations with both parents for grain quality traits (GL, GW, and % chalk) and their correlations under HNT stress, identify the QTLs with SNP markers by using Inclusive Composite Interval mapping (IM-Add and IM-EPI) method, validate the QTL-linked markers with previously reported QTLs, and characterize the QTL regions to identify the candidate genes for grain quality traits with HNT stress tolerance using genome-scale and transcriptome analyses. This novel study of molecular mapping and characterization of these QTLs at genome and transcriptome levels will be useful to provide insights on favorable allele mining, identification of potential candidate genes related to grain quality and HNT stress tolerance mechanisms in US grown *TRJ* rice cultivars, which would be beneficial to rice breeders and geneticists for developing good grain quality and heat tolerant rice cultivars in the US.

## Materials and methods

### Plant material and growth conditions

The MY2 population of 185 *F*_*5*_-derived bulk RILs (F_12_) developed from a cross between two released elite US long grain rice varieties, “Cypress and LaGrue”, was developed as part of the RiceCap project^68^. Cypress is tolerant to heat stress and good grain quality (low chalky) under HNT stress while LaGrue is highly sensitive to heat stress and poor grain quality (high chalky) under HNT stress^11,69^. The MY2 RIL population was originally obtained from the USDA ARS Dale Bumpers National Rice Research Center, Stuttgart, AR, United States and each line was purified using single plant (F_10_) lines and further re-derived from single panicle in the 2016-field trial at H. Rouse Cafey Rice Research Station, Louisiana State University (LSU) Agricultural Center, Rayne, LA 70578, USA. The permissions from the USDA and LSU Agricultural Center, LA, USA were obtained to collect and use the MY2 RIL population for this study.

A sample of ~ 30 seeds from each RIL along with both parents was germinated in single plastic pots, of size 15 cm × 15 cm, filled with a mixture of the SunGro professional potting mix (Sun Gro Horticulture Distribution, Agawam, MA, United States) and field soil, and grown in the greenhouse at the Harry R. Rosen Alternative Pest Control Center at the University of Arkansas, Fayetteville, AR, USA^70^. At 10 days after germination, equal-sized seedlings of each RIL and both parents were transplanted in 3-gallon-plastic pots filled with a mixture of potting mix and field soil. The RILs and parents were then grown in the greenhouse until booting stage (R2). For greenhouse conditions, the temperature was set to 30 ± 1 °C (86 ± 1 °F) during the day and 22.2 ± 1 °C (72 ± 1 °F) at night^71^. The light was set to a light/dark 13/11-h cycle with maximum photo-synthetically active radiant (800–1000 μmol PAR m^−2^ s^−1^) light and 60–65% relative humidity (RH) for the growth of the rice plants. The experimental design was a completely randomized design (CRD) with three replications (each replication is one plant in the pot). The plants were checked for water every day and fertilized with the Peter Professional soluble fertilizer (Allentown, PA, USA) containing chelated iron once a week for full vegetative growth. Plant protection methods were applied to prevent insect pests and diseases, which followed the Rosen Center (University of Arkansas, Fayetteville, AR, USA) greenhouse standard procedures.

### Phenotypes and high nighttime temperature (HNT) stress treatment

**Figure 1:**
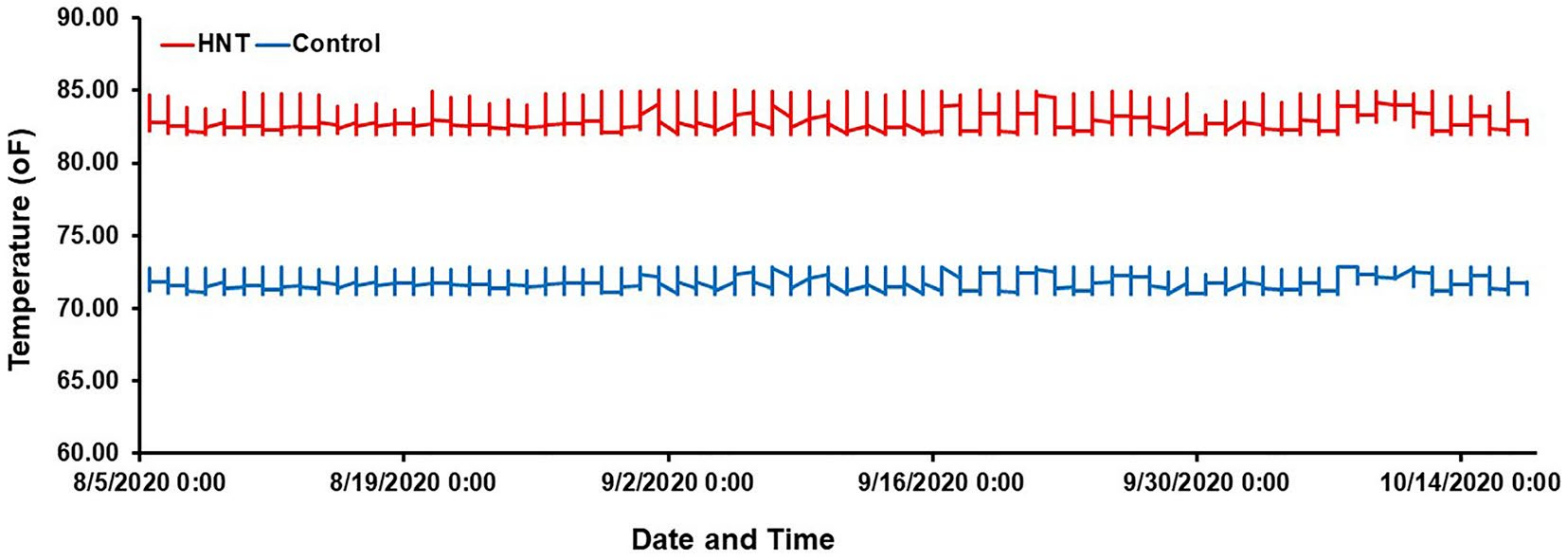
Experiment comparing high nighttime temperature (HNT) stress with control treatments on 185-MY2 RIL population with both parents “Cyress and LaGrue”. The nighttime temperatures were recorded during the screen of the population. The nighttime temperature during each night was plotted across the duration of the experiment for the HNT stress and control treatments, the red line plots the HNT stress temperature treatment, and the blue line is the plot of the control treatment at nighttime. The HNT stress temperature was stably maintained at 82.4 ± 1 °F (28 ± 1 °C), while the control treatment was also held steady at 72 ± 1 °F (22.2 ± 1 °C) during the experiment until harvest maturity.

At booting stage (R2), as described by Counce et al.^72^, three major panicles per plant were tagged in each RIL and both parents. The plants with tagged panicles were then moved to the greenhouse with the HNT stress treatment that was maintained at a day/night temperature of 30 °C (86 °F)/28 °C (82.4 °F) for 10 h (20:00–6:00) while the control treatment was set at a day/night temperature of 30 °C (86 °F)/22.2 °C (72 °F) until harvest maturity (approximately 18–20% grain moisture content). The HOBO data loggers/sensors (Onset HOBO^®^ data logger, Cape Cod, MA, US) were installed in both greenhouses (Control and HNT stress treatments) for the continuous monitoring and recording of the day and nighttime temperatures until physiological maturity (Fig. 1). The data logger system was operated by the HOBOware^®^ Pro software/app with compatible devices. At harvest maturity, all the panicles (under Control and HNT stress treatments) of each RIL and both parents were collected separately in individual brown bags, air-dried (12–14% grain moisture content), and used for the phenotyping of grain quality traits such as GL, GW, and %chalk. For grain quality estimation, rough rice grains of each plant of the RIL and both parents were de-hulled using a manually operated dehuller (Rice Husker TR120, China). The GL, GW, and %chalk were estimated in 100-brown (dehulled) rice grain samples twice in each replicate of the RIL and parents, using an image analysis system WinSeedle™ Pro 2020a (Regent Instruments Inc., Sainte-Foy, Quebec, Canada) and expressed as percent of affected grains in the projected area.

### Statistical analysis and phenotypic evaluations

The statistical analysis of the phenotype data was performed using R statistical packages *v4.1.0* and John’s Macintosh Project (JMP) Genomics Pro version 12.0 for descriptive statistics. The test for normal distribution and homogeneity of variances was done using the Shapiro–Wilk test and Brown–Forsythe test, respectively. An analysis of variance (ANOVA) was carried out with a statistical model that included the effects of RIL, treatment (control and HNT stress), and the interaction between RIL and treatment. The Tukey’s Honest Significant Difference (HSD) test was used to compare the means of treatments among all the RILs for significant effects (Tukey’s HSD, *p* < 0.05) using the *hsd* function in R packages and JMP version 12, as Tukey’s HSD can determine slight differences between the means. Pearson’s correlation coefficient was carried out between grain quality traits under control and HNT stress conditions using the R package ‘*corrplot*’. The broad-sense heritability (*H*^2^) was estimated to describe how the environment affected GL, GW and %chalk in the RIL population using the *lmer4* function in R v4.1.0^73,74^. The confint function^73^ was used to compute the standard errors of the variance estimates provided by *lmer*, and these were then proliferated to use the 95% confidence intervals for the *H*^2^.

### Genotyping and construction of genetic map

The MY2 RIL population with both parents was genotyped with 1K-Agriplex platform on single plant re-derived from single panicle in the field trial. In addition, allele-specific primer (KASP) data was generated to genotype the RIL population to be more informative genetically. For genotyping of the MY2 RIL population, DNA was extracted from the young leaf tissue of each individual plant of each RIL using a modified CTAB method^75^, and KASP assays were designed by LGC genomics^76^. The SNPs with monomorphic, heterozygous, and more than 30% missing data, were removed and a set of 1178 (1042 SNPs + 136 KASP) markers was used for further genetic analysis: QTL analysis and construction of genetic and physical linkage maps. The genetic map was constructed using 1178 SNP markers with MAP functionality of QTL IciMapping *v4.2* software^77^. Map has three basic steps: grouping, ordering, and rippling. Grouping of binned markers was performed using logarithms of the odds (LOD) threshold value, resulting in assembling 12 linkage groups representing the 12 chromosomes of rice. The RECORD (Recombination Counting and ORDering) algorithm was performed to order 1178 SNP markers within the linkage groups over 12 chromosomes. Recombination frequencies between markers were converted into centiMorgan (cM) by using the Kosambi mapping function. The physical map was drawn with 1178 SNP markers with their physical positions (extracted from IRGSPv1.0: http://rice.uga.edu/) using MapChart 2.0 software^78^.

### Quantitative trait loci (QTL) analysis

QTL analysis was performed using IciMapping v4.2 software^77^ with the BIP functionality (https://www.isbreeding.net/) in the MY2 RIL population for grain quality traits. The significant LOD threshold to declare the significant QTLs was calculated using 1000 permutations and Type I error rate (*p* < 0.05). To detect the putative additive and epistatic QTLs, the Inclusive Composite Interval Mapping (ICIM-ADD) and Epistatic QTL (ICIM-EPI) functions were performed, respectively as mapping methods. The mapping parameters were 1.0 cM scanning steps and a probability of 0.01 in stepwise regression for additive QTL identification. For the detection of epistasis, scanning steps of 5.0 cM, a probability of 0.0001 in stepwise regression, and a LOD threshold of 4.0 or more were used to declare significant epistasis. QTLs explaining ≥ 10.0% phenotypic variance with ≥ 2.5 LOD were declared significant. The MapChart v2.0 software^78^ was used for graphical representation of QTLs on the rice chromosomes.

### Allele mining for the QTL regions in a japonica diversity panel

The whole genomes of 83 rice accessions (Supplementary Table S1) comprising the *japonica* diversity panel (JDP) of the USDA mini-core rice collection (URMC) were sequenced by Novogene (www.en.novogene.com), with an average coverage of approximately 20×^16^. The raw FASTQ reads from the 83 genomes were mapped to the reference rice genome cv. Nipponbare (IRGSP 1.0), using the Burrows–Wheeler Aligner (BWA), and the bam files were used to call SNPs using GATK^79^. Approximately 6.1 million high quality SNPs were retained (less than 2% missing rate and more than 5% minor allele frequency) and annotated. Of these, the SNPs present in 15 QTLs regions were selected and further analyzed for natural genetic variation. These 15 QTLs representing cumulative 51 Mb region of the genome spread across nice chromosomes, which analyzed further. This SNP dataset was further filtered to retain the SNPs that were polymorphic between both parents “Cypress and LaGrue”. The chromosome wide distribution of the various genomic features including QTLs and SNPs was visualized in circos^80^.

### Characterization of the QTL regions and Identification of candidate genes using genome and transcriptome scale analyses

We identified candidate genes involved in GL, GW and % chalk under HNT stress by integrating QTLs and the expression QTL (eQTL) analyses of both parents “Cypress and LaGrue”. RNA-Seq data from caryopsis tissues at R6 stage (milky endosperm) under control and HNT stress conditions with replicates were used for differential gene expression analyses using the *DESeq2* R package^81^. The differentially expressed genes (DEGs) were investigated using the GxE model on the ratio Cypress-HNT vs. Cypress-Control / Lagrue-HNT vs. Lagrue-Control to understand the effect of HNT stress in a genotype-dependent manner^82^. Genes with threshold value of log_2_ fold change ≥ 1 and *padj* < 0.05 were assigned as DE and used for subsequent analyses. All the experiments on rice plants were performed in accordance with relevant guidelines and regulations.

## Results

### Phenotypic variation and trait correlation

**Figure 2:**
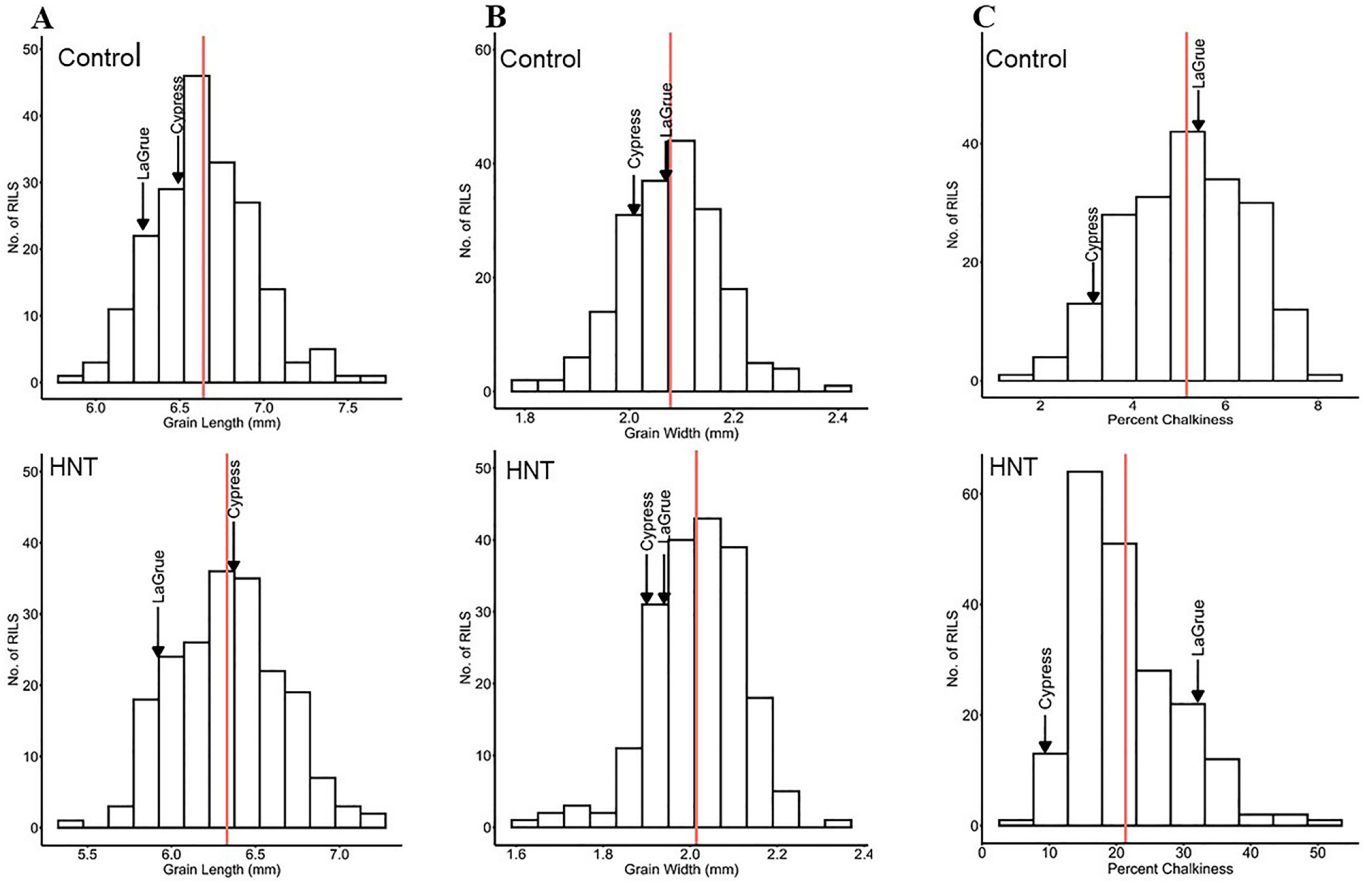
Frequency distribution showing broad range of variation for grain quality traits under control and high nighttime temperature (HNT) stress conditions. (**A**) Grain length (GL-mm) in response to control (top) and HNT stress (bottom). (**B**) Grain width (GW-mm) response to control (top0 and HNT stress (bottom). (**C**) Percent chalkiness showing response to control (top) and HNT stress (bottom). Vertical line (red) indicates the mean of the recombinant inbred lines (RIL) population. Bottom headed arrows showed the mean of both parents “Cypress and LaGrue”.

**Figure 3:**
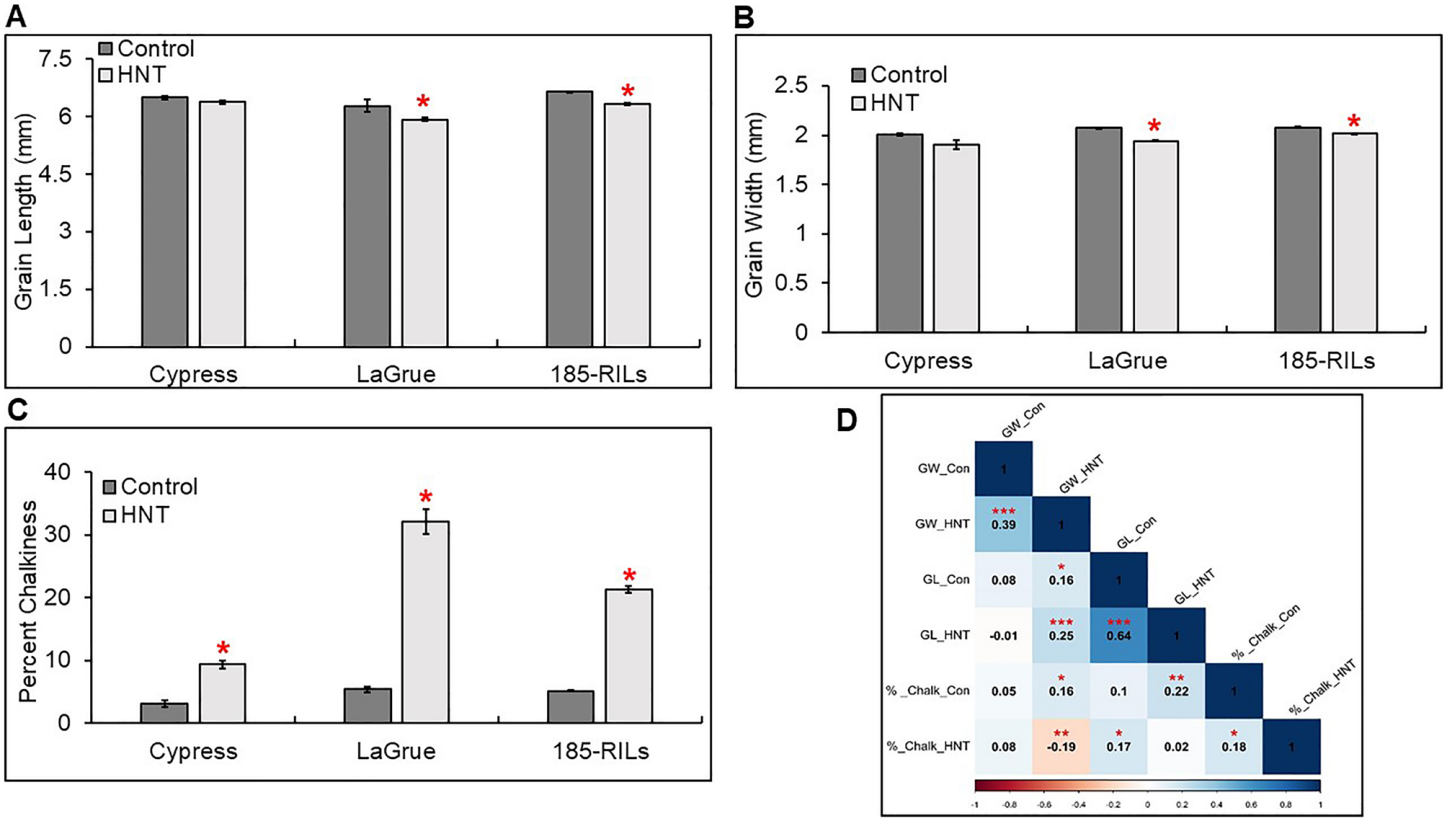
Bar plots showing the phenotypic variation in grain quality traits in 185-MY2 RIL population with both parents, “Cypress and LaGrue”, under high nighttime temperature (HNT) stress condition compared to control condition. (**A**) Grain length (mm) response to HNT stress compared to control condition in the RIL population and both parents. (**B**) Grain width (mm) response to HNT stress compared to control condition in the RIL population and both parents. (**C**) Percent chalkiness response to HNT stress compared to control condition in the RIL population and both parents. (**D**) Pearson’s correlation coefficient between the grain quality traits (grain length-GL, grain width-GW, and percent chalkiness-%chalk). *p < 0.05, **p < 0.01, ***p < 0.001 indicate significant correlations among traits.

To evaluate the effects of HNT stress compared to control conditions (Fig. 1), the MY2 RIL populations with both parents, “Cypress and LaGrue” were analyzed and showed a wide range of phenotypic variation in gain quality traits such as GL, GW, and %chalk. Frequency distribution and histograms of all the grain quality traits indicate distribution among the RIL populations under control and HNT stress conditions. Values of each grain quality trait among the RIL populations extended beyond both parents show transgressive variation under control and HNT stress conditions (Fig. 2A–C, Table 1), indicating that alleles with positive and negative effects were derived from both parents. A wide range of phenotypic variation for all the grain quality traits under control and HNT stress conditions are summarized in Table 1 and indicates, cumulative action of many genes, quantitative inheritance. The extended variation, explained by the percent genetic variation (PGV), was observed in the RIL populations, showing 29.51%, 32.84%, and 215.04% genetic variation in GL, GW, and % chalk under HNT stress, respectively, while 27.41%, 28.85%, and 122.28% genetic variation in GL, GW, and % chalk under control treatment, respectively (Table 1). The parents, Cypress and LaGrue, were significantly different for GL, GW, and % chalk under HNT stress as LaGrue shows higher reduction in GL, GW and increase in % chalk, showing HNT stress sensitivity compared to Cypress that shows HNT stress tolerance under HNT stress conditions (Fig. 3A–C). High broad sense heritability (*H*^2^), ranging from 0.587 to 0.897, was observed for GL, GW, and %chalk in the RIL populations under control and HNT stress treatments (Table 1). Under HNT stress, the RIL populations show higher *H*^*2*^ (0.702–0.897) compared to control condition, ranging from 0.587 to 0.657, for GL, GW, and %chalk.

**Table 1:** Descriptive statistics of phenotypic data of grain quality traits of 185 recombinant inbred lines (RILs) with both parents (Cypress and LaGrue) evaluated under control and high nighttime temperature (HNT) stress conditions.

Descriptive statistics	Genotype	Treat	GL (mm)	GW (mm)	%Chalk
Mean	Cypress	Control	6.49	2.01	3.14
		HNT	6.37	1.9	9.36
	LaGrue	Control	6.28	2.07	5.41
		HNT	5.92	1.94	32.09
	RILs	Control	6.64	2.08	5.16
		HNT	6.33	2.01	21.34
Range	RILs	Control	1.82	0.6	6.3
		HNT	1.86	0.702	45.89
Standard deviation	Cypress	Control	0.082	0.023	0.82
		HNT	0.057	0.077	1.09
	LaGrue	Control	0.276	0.015	0.84
		HNT	0.071	0.018	3.472
	RILs	Control	0.152	0.046	0.84
		HNT	0.128	0.048	2.17
Variance	Cypress	Control	0.006	0.0005	0.67
		HNT	0.003	0.005	1.19
	LaGrue	Control	0.076	0.0002	0.7
		HNT	0.005	0.0003	1.02
	RILs	Control	0.041	0.003	1.05
		HNT	0.031	0.004	1.25
Coefficient of variation (%)	Cypress	Control	1.27	0.77	26.25
		HNT	0.9	0.95	11.68
	LaGrue	Control	4.39	1.13	15.54
		HNT	1.21	3.96	10.81
	RILs	Control	2.3	2.25	16.26
		HNT	2.03	2.42	10.19
Percent genetic variation (PGV)	RILs	Control	27.41	28.85	122.28
		HNT	29.51	32.84	215.04
Skewness	RILs	Control	0.215	− 0.077	− 0.227
		HNT	0.153	− 0.522	0.9545
Kurtosis	RILs	Control	0.338	0.533	− 0.6841
		HNT	− 0.094	0.285	1.023
W-test	RILs	Control	0.9876	0.9915	0.9657
		HNT	0.9851	0.977	0.9389
Broad sense heritability (*H*^2^)	RILs	Control	0.657	0.651	0.587
		HNT	0.762	0.702	0.897

*Treat* treatment conditions (Control and HNT stress), *GL (mm)* grain length in millimeter (mm), *GW (mm)* grain width in millimeter (mm), and *%chalk* percent chalkiness.

The trait correlation analysis was performed to determine the relationship between %chalk, GL, and GW in the RIL populations under control and HNT stress conditions (Fig. 3D). The %chalk shows positive correlation with GL (*r* = 0.1) and GW (*r* = 0.05) under control condition while it exhibits significant positive correlation with GL (*r* = 0.17, *p* < 0.05) and negative correlation with GW (*r* = − 0.19, *p* < 0.01) under HNT stress condition. The correlation of GL with GW under control condition is positive correlation (r = 0.08) while under HNT stress, it shows significant positive correlation (*r* = 0.25,* p* < 0.001).

### Genetic map construction

**Figure 4:**
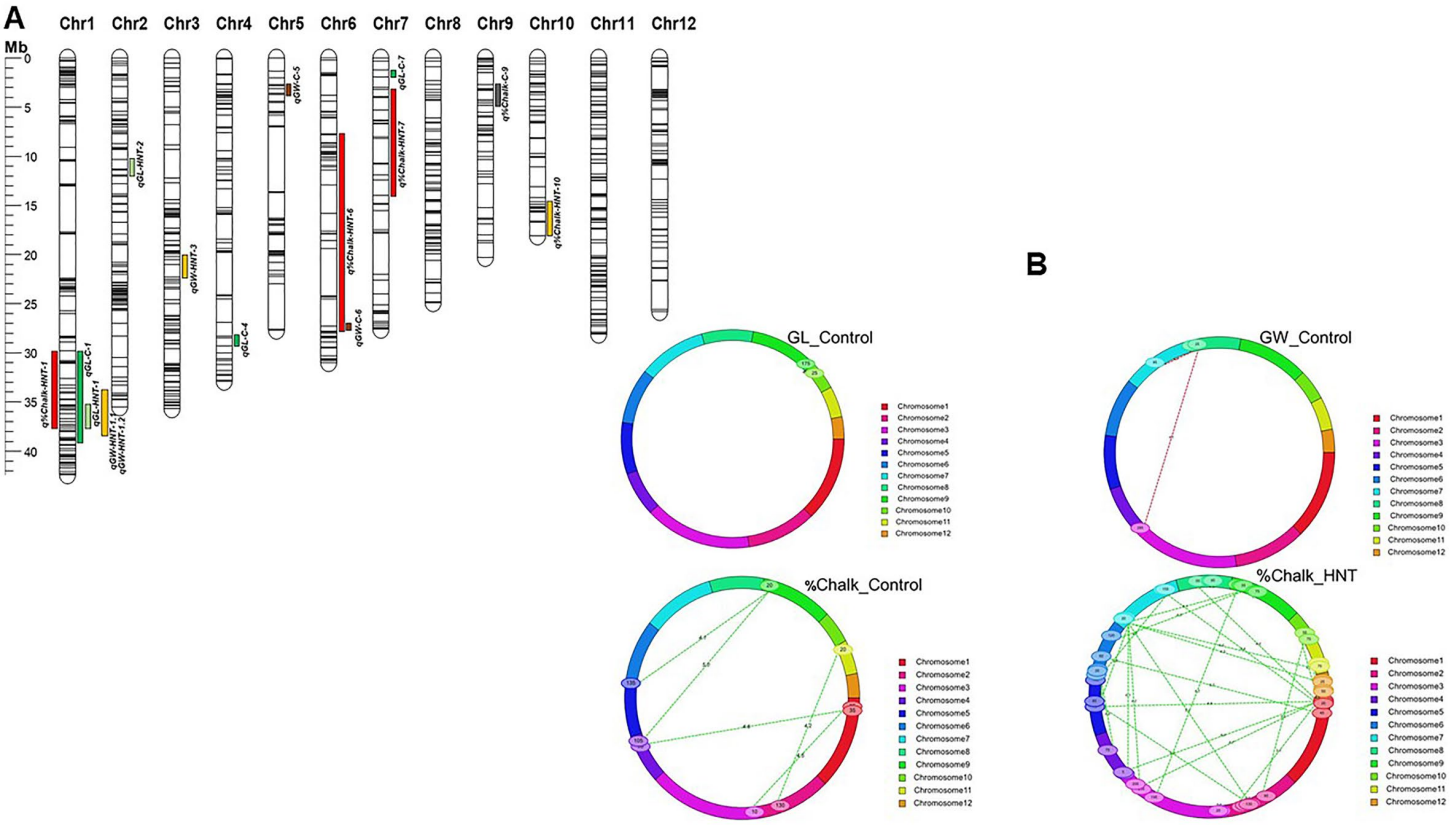
Graphic representation of molecular mapping of quantitative trait loci (QTLs) for grain quality traits with physical position of SNPs on rice chromosomes and QTL × QTL interaction in the MY2 RIL population. (**A**) 15 QTLs (6 QTLs under control and 9 QTLs under HNT stress conditions) associated with grain quality traits such as grain length (GL-mm), grain width (GW-mm), and percent chalkiness (% chalk) identified by ICIM mapping. (**B**) Circular illustrations of epistatic quantitative trait loci (QTLs) of grain quality traits (GL, GW, and % chalk) in the MY2 RIL population under control and high nighttime temperature (HNT) stress conditions. The dotted lines indicate SNP marker pairs interacting significantly on same or different chromosomes with their corresponding LOD value due to epistatic effect.

The genetic map was constructed using 1178 polymorphic SNP markers containing a total length of 1897.87 cM with an average maker density of 1.6 markers per cM in the rice genome. The marker information and marker position (in cM) on each chromosome are shown in Supplementary Fig. S1. The number of SNPs per chromosome ranged from 54 SNPs on chromosome 10 to 157 SNPs on chromosome1. More than 80% SNP markers show a distance of 3.0 cM or less between adjacent markers in the map. Several gaps more than 20 cM between markers were shown on different chromosomes with the largest gaps where recombination is less frequent. To fill these gaps, allele mining in the QTL regions would be helpful to enhance the SNP markers resolution on the map for further genetic analyses. The physical position of each SNP marker across the rice genome based on their genomic position is shown in Fig. 4A.

### QTL mapping and QTL x QTL interaction

A total of 15 additive QTLs associated with grain quality traits (GL, GW, and % chalk) showing major and minor effects were identified in 185-RIL populations under control and HNT stress conditions, using BIP functionality of CIM mapping approach with 1178 SNP markers (Table 2, Fig. 4A, Supplementary Figs. S2, S3). Out of these 15 additive QTLs, a total of 6 QTLs, with additive effects ranging from − 0.99 to 0.0267 and individually explained by 3.33 to 8.27% of the phenotypic variation, were identified for GL, GW and % chalk, under control condition (Fig. 4A, Supplementary Fig. S2A–C), on six chromosomes (chr1, chr4, chr5, chr6, chr7, and chr9) while under HNT stress condition, 9 QTLs, with additive effects ranging from − 8.8 to 0.028 and individually explained by 6.39 to 51.53% of the phenotypic variation, were detected for GL, GW, and % chalk on six chromosomes (chr1, chr2, chr3, chr6, chr7, and chr10) in rice genome (Table 2, Fig. 4A, Supplementary Fig. S3A–C).The additive effect is explained as one-half of the difference between the average effects of parental alleles (Cypress and LaGrue). In the results, a negative additive effect indicates that the favorable allele was contributed by Cypress; whereas a positive additive effect suggests that LaGrue added the favorable allele. A QTL was considered as major effect when the phenotypic variation explained in percentage (PVE %) was more than 20%^83^. The details information of all the QTLs under control and HNT stress conditions are shown in Table 2. On physical map, the average distance between left and right markers flanking QTLs was less than 1.0 Mb and had a range of 157,670 bp to 19,092,637 bp (Fig. 4A, Supplementary Figs. S2, S3).

**Table 2:** Quantitative trait loci (QTLs) associated with grain quality traits (GL, GW, and % chalk) under control and high nighttime temperature (HNT) stress conditions in the MY2 RIL population derived from the cross of two U.S. rice cultivars (Cypress and LaGrue) using ICIM mapping.

Treat	QTL	Chr	Pos (cM)	Left flaking marker		Right flaking marker		LOD	PVE (%)	Add	Fav. Allele
				Marker	Pos (cM)	Marker	Pos (cM)				
Control	*qGL-C-1*	1	113	SNP0129_129	110	SNP0130_130	114	4.78	8.274	− 0.11	Cypress
	*qGL-C-4*	4	102	SNP1179_1179	101.5	SNP386_4	102.5	3.51	5.469	− 0.082	Cypress
	*qGL-C-7*	7	73	SNP405_7	67.5	SNP827_7	87.5	2.61	4.841	− 0.077	Cypress
	*qGW-C-5*	5	19	SNP399_5	17.5	SNP0511_511	21.5	3.21	7.634	0.0267	LaGrue
	*qGW-C-6*	6	152	SNP0638_638	149.5	SNP333_6	154	3.25	7.441	0.0264	LaGrue
	*q%Chalk-C-9*	9	55	SNP1170_1170	33.5	SNP0829_829	68.5	3.32	3.325	− 0.996	Cypress
HNT	*qGL-HNT-1*	1	107	SNP0118_118	101.5	SNP291_1	107.5	4.85	10.453	− 0.12	Cypress
	*qGL-HNT-2*	2	104	SNP0212_212	97	SNP506_2	105.5	2.93	6.389	− 0.081	Cypress
	*qGW-HNT-1.1*	1	86	SNP0108_108	83.5	SNP0109_109	89.5	3.79	9.2892	− 0.033	Cypress
	*qGW-HNT-1.2*	1	88	SNP0109_109	87.73	SNP0112_112	89.18	3.56	8.0977	− 0.031	Cypress
	*qGW-HNT-3*	3	135	SNP310_3	132.5	SNP0350_350	139.5	2.83	6.4213	0.028	LaGrue
	*q%Chalk-HNT-1*	1	12	SNP1203_1203	6.5	SNP99_1	16.5	4.33	49.874	− 7.273	Cypress
	*q%Chalk-HNT-6*	6	98	SNP0603_603	73.29	SNP0640_640	107.5	4.86	51.47	− 7.294	Cypress
	*q%Chalk-HNT-7*	7	12	SNP1163_1163	1.09	SNP0672_672	17.5	4.42	51.527	− 7.259	Cypress
	*q%Chalk-HNT-10*	10	70	SNP0923_923	69.5	SNP212_10	72.5	2.61	28.775	− 8.785	Cypress

*Trt* treatment conditions (control and HNT stress), *QTL* quantitative trait locus, *Chr* chromosome in the rice genome, *Pos(cM)* genetic position in centiMorgan (cM) on the linkage map, *LOD* logarithm of the odds peak/score was set ≥ 2.5 as threshold for detecting significant QTL in the mapping, *PVE(%)* total phenotypic variance explained by the QTL in percentage (%), *Add* additive effect values indicate the direction of favorable allele from the parents, *Fav.allele* Favorable allele showing the negative additive effect value that means the direction of favorable allele from Cypress, while showing positive additive effect value pointing the direction of the favorable allele from LaGrue.

Under control condition, three QTLs for GL, *qGL-C-1*, *qGL-C-4*, and *qGL-C-7*, were detected with LOD scores of 4.78, 3.51, and 2.61 and showed PVE (%) of 8.27, 5.469, and 4.84%, respectively. The size of *qGL-C-1*, *qGL-C-4*, and *qGL-C-7* QTLs was 4.0 cM, 1.0 cM, and 20.0 cM within flanking SNP makers, SNP0129_129 (38,652,270 bp) & SNP0130-130 (39,278,883 bp), SNP1179_1179 (29,114,708 bp) & 29,299,845 bp), and SNP405_7 (2,036,558 bp) & SNP827_7 (2,721,936 bp) on chromosomes 1, 4, and 7, respectively (Table 2, Fig. 4A, Supplementary Fig. S2A). For GW, two QTLs, *qGW-C-5* and *qGW-C-6*, were identified with LOD scores of 3.21 and 3.25, and exhibited PVE (%) of 7.63% and 7.44%, respectively. The size of these QTLs, *qGW-C-5* and *qGW-C-6,* was 4.0 cM and 4.5 cM within flanking SNP markers, SNP399_5 (3,709,209 bp) & SNP0511_511 (4,129,551 bp) and SNP0638_638 (28,004,251 bp) & SNP333_6 (28,688,881 bp) on chromosomes 5 and 6, respectively (Table 2, Fig. 4A, Supplementary Fig. S2B). For % chalk, the QTL, *q%chalk-C-9*, was detected with LOD score of 3.32 and showed PVE (%) of 3.33%. The size of the QTL was 35 cM within flanking SNP markers, SNP1170_1170 (4,052,650 bp) and SNP0829_829 (6,667, 280 bp) on chromosome 9 (Table 2, Fig. 4A, Supplementary Fig. S2C). Under HNT stress conditions, for GL, two QTLs, *qGL-HNT-1* and *qGL-HNT-2*, were identified with LOD scores of 4.85 and 2.93 and showed PVE (%) of 10.45% and 6.40%, respectively. These QTLs were sized of 6.0 cM (1,936,656 bp) and 8.5 cM (613,935 bp) within flanking SNP markers, SNP0118_118 (36,107,132 bp) & SNP291_1 (38,043,788 bp) and SNP0212_212 (11,707,132 bp) & SNP506_2 (12,321,173 bp) on chromosomes 1 and 2 respectively (Table 2, Fig. 4A, Supplementary Fig. S3A). For GW, three QTLs, qGW-HNT-1.1, qGW-HNT-1.2, and qGW-HNT-3, were identified with LOD scores of 3.79, 3.56, and 2.83, and showed PVE (%) of 9.28%, 8.1%, and 6.5%, respectively. These three QTLs were sized of 6.0 cM (402,983 bp), 1.45 cM (157,670 bp), and 7.0 cM (214,274 bp) within flanking SNP markers, SNP108_108 (34,274,861 bp)&SNP109_109(34,677,844 bp),SNP109_109(34,677,844b) &SNP0112_112(34,835,514 bp), and SNP310_3 (22,796,525 bp) & SNP350_350 (23,010,799 bp) on chromosomes 1, 1, and 2, respectively (Table 2, Fig. 4A, Supplementary Fig. S3B). Four QTLs for %chalk, *q%chalk-HNT-1*, *q%chalk-HNT-6*, *q%chalk-HNT-7*, and *q%chalk-HNT-10*, were detected with LOD scores of 4.33, 4.86, 4.42, and 2.61 and explained phenotypic variation (%) of 49.87%, 51.47%, 51.53%, and 28.77%, respectively. The size of these four QTLs was 10.0 cM (7,919,913 bp), 34.2 cM (19,092,637 bp), 16.4 cm (11,701,288 bp) within flanking SNP markers, SNP1203_1203 (30,498,826 bp) & SNP99_1 (38,418,739 bp), SNP1163_1163 (4,001,958 bp) & SNP0672_672 (15,703,246 bp), and SNP0923_923 (17,908,351 bp) & SNP212-10 (21,817,967 bp) on chromosomes 1, 6, 7 and 10, respectively (Table 2, Fig. 4A, Supplementary Fig. S3C).

The polygenic behavior of grain quality traits (GL, GW, and %chalk) may identify many QTLs with smaller effects and epistasis between distinguish loci in the genome. Several QTLxQTL interactions were detected between different loci for each grain quality trait under individual control and HNT stress conditions (Supplementary Table S2, Fig. 4B). To declare a significant epistatic interaction, QTL x QTL interaction with a threshold LOD > 4.0 was used^84^. Under control conditions, loci associated with %chalk on chr1 (30.49–38.49 mb), chr2 (17–18.97 mb), chr4 (27.70–29.29 mb), and chr5 (22.63–23.97 mb) showed epistatic interactions with loci on chr3 (2.87–3.39 mb) & chr4 (30.15–33.15 mb), chr11 (16.41–17.36 mb), and chr9 (4.06–6.67 mb), respectively exhibiting PVE range 0.51% to 2.37% with epistatic effect range − 8.23 to 5.34 (Supplementary Table S2, Fig. 4B). Loci associated with GL on chr3 (5.89–27.14 mb) and chr7 (2.73–4.74 mb) interacted with the loci (17.63–21.87 mb and 6.27–9.21 mb) on chr8 showing PVE 2.79% and 2.68% and epistatic effect 0.134 and 0.109, respectively. For GW, the epistatic interaction was observed between loci on chr9 (17.74–18.82 mb) and the loci on chr10 (11.93–13.43 mb) with PVE 1.85% and epistatic effect 0.036 (Supplementary Table S2, Fig. 4B). Under HNT stress conditions, for GL and GW, no interaction was detected. For % chalk, locus on chr1 (30.50–38.42 mb) showed significant epistatic interaction with loci on chr2 (21.33–22.03 mb), chr3 (7.35–31.57 mb), chr4 (16.29–16.33 mb), chr5 (6.61–14.64 mb), chr6 (0.36–9.98 mb), chr7 (18.63–25.95 mb), chr8 (22.62–24.46 mb), chr10 (13.72–16.83 mb), chr11 (17.59–27.83 mb), and chr12 (22.75–25.68 mb) showing PVE range from 0.62 to 2.37% and epistatic effect range − 6.06 to 1.31. In addition, loci on chromosomes 2, 4, 5, 6, and 7 showed significant interactions with loci on several chromosomes showing PVE range 0.50% to 2.79% and epistatic effect range − 8.23 to 5.39 (Supplementary Table S2). The details information about QTL × QTL interaction is shown in Supplementary Table S2.

### Co-localization of QTLs with previously reported QTLs

There has been no bi-parental mapping for grain quality traits in tropical *Japonica* background under HNT stress reported. In order to validate these identified QTLs in this study, we investigated the co-localization of all the detected QTLs related to GL, GW, and % chalk under control and heat stress conditions with 15 previously reported studies worldwide in *japonica* rice background^21,46,51,55,62,85–94^. The 15 independent publicly available mapping studies, published between 2001 to 2021, reported 29 major effect QTLs related to GL, GW, ratio of grain length and width (RLW), and chalkiness related components such as percent chalkiness (PC), degree of chalky endoderm (DEC), chalk rate (CR), percent grain white chalk (PGWC), white kernel (WK), white back (WB), back chalk (BCHK), percent white back (PWB), percent grain chalkiness (PGC), and chalky kernel (CK), were downloaded and used for extraction of the genome position of the QTLs for co-localization analysis (Table 3). The QTLs, *qGL-C-1* under control condition and *qGL-HNT-1* under HNT stress on chr1 related to GL, were co-localized with previously reported major effect QTL *GL1-4*^85^ in the genomic region between 36,107,132 and 39,278,883 bp in the genome. Under control conditions, the *QTLs, qGW-C-5* on chr5 and *qGW-C-6* on chr6 for GW, were co-localized with three previously reported major effect-QTLs (*gGL5-3*, *qGW-5*, and *qGW5*)^21,46,85,86^ in the genomic region (3,709,209–4,129,551 bp) on chr5 and two major QTLs (*qGW6-7 and qRLW-6*)^85,86^ in the genomic region (28,004,251–28,688,881 bp) on chr6, respectively. The QTL, *q%chalk-C-9* for % chalk under control conditions, was coincided in the genomic region (4,052,650–6,667,280 bp) with three well-known and major effect-QTLs, *qPC9.1, qDEC-9*, and *qCR9*, on chr9^46,87,88^.

**Table 3:** Previously reported quantitative trait loci (QTLs) co-localized with the genomic regions of 15 QTLs related to grain quality traits (GL, GW, and % chalk) under control and high nighttime temperature (HNT) stress.

Treat	QTL	Chr	Left flaking marker	Right flaking marker	QTL interval (bp)	Co-localization with pre reported QTLs	References
Control	*qGL-C-1*	1	SNP0129_129	SNP0130_130	38,652,270–39,278,883	*qGL1-4*	^85^
	*qGL-C-4*	4	SNP1179_1179	SNP386_4	29,114,708–29,299,845	–	–
	*qGL-C-7*	7	SNP405_7	SNP827_7	2,036,558–2,721,936	–	–
	*qGW-C-5*	5	SNP399_5	SNP0511_511	3,709,209–4,129,551	*qGL5-3, qGW-5, qGW5*	^21,46,85,86^
	*qGW-C-6*	6	SNP0638_638	SNP333_6	28,004,251–28,688,881	*qGW6-7, qRLW-6*	^85,86^
	*q%Chalk-C-9*	9	SNP1170_1170	SNP0829_829	4,052,650–6,667,280	*qPC9.1, qDEC-9, qCR9,*	^46,87,88^
HNT	*qGL-HNT-1*	1	SNP0118_118	SNP291_1	36,107,132–38,043,788	*qGL1-4*	^85^
	*qGL-HNT-2*	2	SNP0212_212	SNP506_2	11,707,238–12,321,173	–	–
	*qGW-HNT-1.1*	1	SNP0108_108	SNP0109_109	34,274,861–34,677,844	*q%Chalk-HNT-1*	–
	*qGW-HNT-1.2*	1	SNP0109_109	SNP0112_112	34,677,844–34,835,514	*q%Chalk-HNT-1*	–
	*qGW-HNT-3*	3	SNP310_3	SNP0350_350	22,79,6525–23,010,799	*qGL3, qGW3*	^89^
	*q%Chalk-HNT-1*	1	SNP1203_1203	SNP99_1	30,498,826–38,418,739	*qGL-HNT-1,qGW-HNT-1.1, qGW-HNT-1.2, qPC1.1, qPGWC-1, qDEC-1b, qWK1-2, qBCHK1-3*	^46,87,90,91^
	*q%Chalk-HNT-6*	6	SNP0603_603	SNP0640_640	10,049,864–29,142,501	*qGW-C-6, qGW6-7, qPC6.1, qRLW-6, qGL-6, gw6, qWB6, qPGWC6.1, qPGWC6.2, qCR6*	^55,62,85, 86,87,89, 92^
	*q%Chalk-HNT-7*	7	SNP1163_1163	SNP0672_672	4,001,958–15,703,246	*qPWB7.1*	^93^
	*q%Chalk-HNT-10*	10	SNP0923_923	SNP212_10	17,908,351–21,817,967	*qPGC-10, qCR10, qCK10, qCK10h, qCD10h*	^51,88,94^

*Trt* treatment conditions (Control and HNT stress), *QTL* 15 quantitative trait loci, *Chr* chromosome in the rice genome.

**Figure 5:**
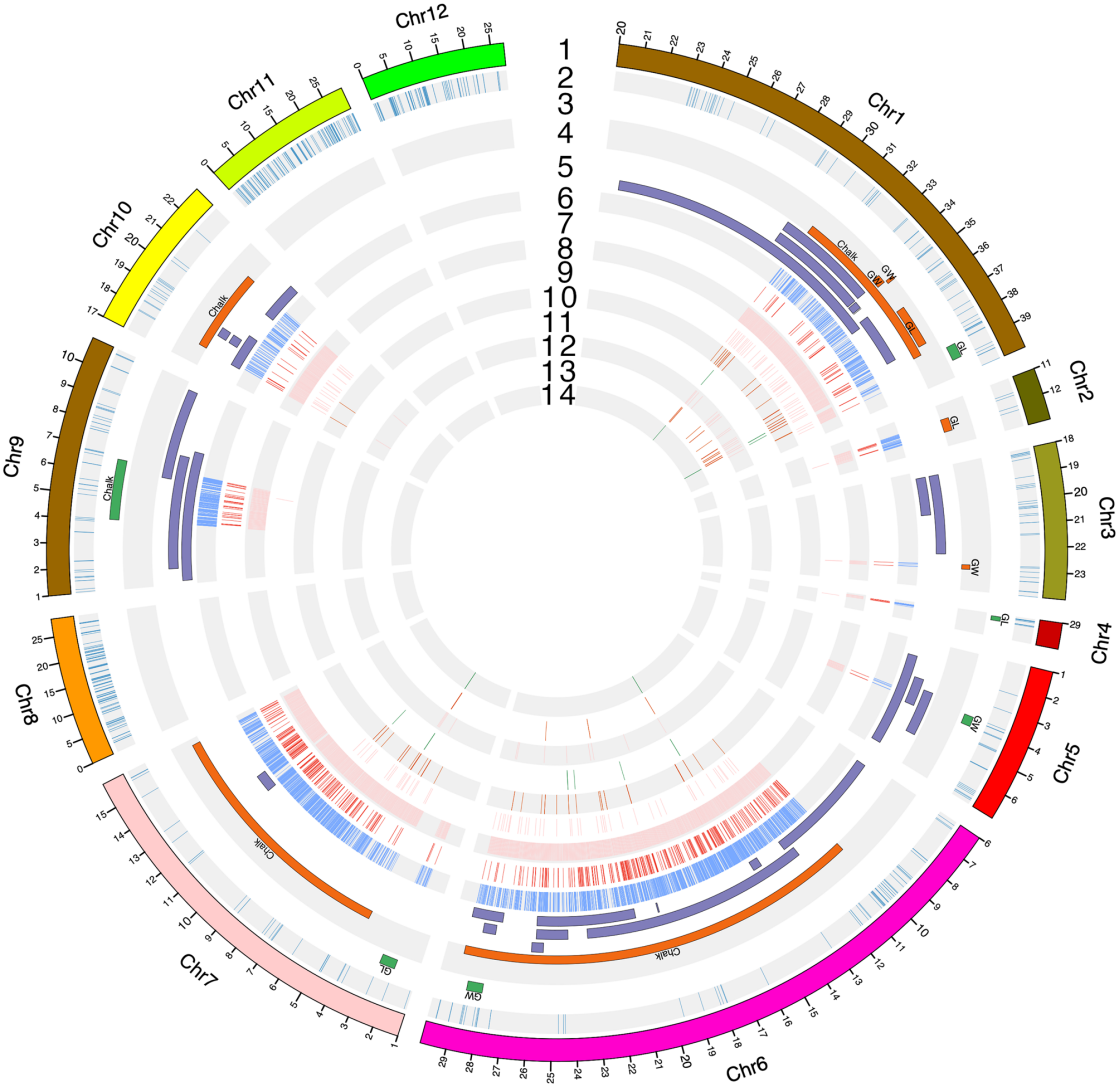
Visual representation of the genomic and transcriptomic characterization of the 51 Mbp genomic region spanning the 15 QTLs. The chromosomes that contain the QTLs and other genomic features are zoomed in for better resolution of the region containing the track information while chromosomes 8, 11 and 12 that lack any genomic information are shown on a regular scale. The tracks are numbered and labeled: 1. Chromosomes, 2. 1178 SNP markers used in QTL mapping, 3. 6 QTLs for grain length, grain width, and percent chalkiness under control condition, 4. 9 QTLs for grain length, grain width, and percent chalkiness under HNT stress condition, 5. Previously reported 29 major effects-QTLs co-localized with the genomic regions of 15 QTLs, 6. High Impact SNPs in the entire QTLs regions identified in 83 genotypes of the Japonica Diversity Panel, 7. High Impact SNPs in the entire QTLs regions, which differentiate Cypress and LaGrue, 8. All genes in the entire QTLs regions, 9. Genes showing differential gene expression only (149 DEGs), 10. High Impact SNPs in DEGs, 11. High Impact SNPs in DEGs that differentiate Cypress and LaGrue, 12. Potential candidate loci/genes of interest (35 genes), 13. High Impact SNPs in the candidate loci/genes, 14. High Impact SNPs in the candidate loci/genes that differentiate Cypress and LaGrue.

Under HNT stress, the QTL, *qGW-HNT-3* on chr3 for GW, was coincided with two previously reported major QTLs, *qGL3* and *qGW3* in the genomic region (22,796,525–23,010,799 bp) on chr3 in rice genome^89^. For % chalk under HNT stress, the QTLs, *q%Chalk-HNT-1* on chr1, *q%Chalk-HNT-6* on chr6, *q%Chalk-HNT-7* on chr7, and q%Chalk-HNT-10 on chr10, were co-localized with prior reported 5 QTLs (*qPC1.1, qPGWC-1, qDEC-1b, qWK1-2, and qBCHK1-3*) in the genomic region (30,498,826–38,418,739 bp) on chr1^47,87,90,91^, 9 QTLs (*qGW6-7,* qPC6.1, *qRLW-6,* qGL-6, gw6, qWB6, qPGWC6.1, qPGWC6.2, qCR6) in the genomic region (10,049,864–29,142,501 bp) on chr6^55,62,85–87,89,92^ , 1 QTL (qPWB7.1) in the genomic region (4,001,958–15,703,246 bp) on chr7^93^, and 5 QTLs (*qPGC-10*, *qCR10, qCK10, qCK10h, qCD10h)* in the genomic region (17,908,351–21,817,967 bp) on chr10^51,88,94^, respectively. The graphic representation of the identified QTLs co-localized with previously reported 29 QTLs in the rice genome is shown in Circos (Fig. 5) and Supplementary Fig. S4.

### Allele mining for the 15 QTL regions in the japonica diversity panel

A set of 83 rice accessions of the JDP was used to mine polymorphic alleles in the 51 Mbp genomic region that contains the 15 QTLs using IRGSP 1.0 as reference genome. A total of 868,823 polymorphic alleles, excluding in/dels and other structural variants, were identified of which 131,629 alleles (15% of total alleles) differentiated both parents, “Cypress and LaGrue” from each other. Based on the functional class of the SNP effects, 55%, 42%, and 3% of these SNPs were classified as missense, silent, and nonsense, respectively. The missense: silent (Mi:Si) ratio for these variants in the genome is 1.3 and the transitions: transversions (Ts:Tv) ratio is 2.6.

According to the SNP annotations, 6160 out of 868,823 SNPs (0.7% of total SNPs) were classified as high impact SNPs (HISs) depending on their type, region, functional class, position, and proximity to the gene and/or gene features. 865 HISs representing 14% of these HISs differentiate the parents, Cypress and LaGrue, further.

The distribution of features found in the QTL regions such as, source markers used to identify the QTLs, genes in QTLs, DEGs, and SNPs associated with each of these features were visualized across the chromosomes (Fig. 5). A summary of the SNPs identified by allele mining in a set of 83 rice accessions of the JDP is presented in the Supplementary Table S3. Assuming the SNPs are uniformly distributed across the cumulative QTL length of 51 Mbp, there is one SNP present every 59 bp for all 83 accessions of the JDP, and one SNP every 389 bp that is polymorphic between the two parents. Similarly, there is one high impact SNP (HIS) every 8.3 Kbp and one Cypress-LaGrue differentiating HIS every 59.1 Kbp.

### Genome and transcriptome scale characterization of the QTL regions and identification of candidate genes for grain quality under HNT stress

A genomic scan of the 51Mbp region spanning the 15 QTLs was performed. Using the IRGSP 1.0 genome feature file (gff3) as a reference, we identified a total of 7117 genes in this region, of which 495 genes were shared between the overlapping QTL regions (*qGW-C-6 & q%Chalk-HNT-6* on chromosome 6, and *qGL-HNT-1, qGW-HNT-1, & qChalk-HNT-1* on chromosome 1). The QTL-wise distribution and order of these genes were also determined.

**Figure 6:**
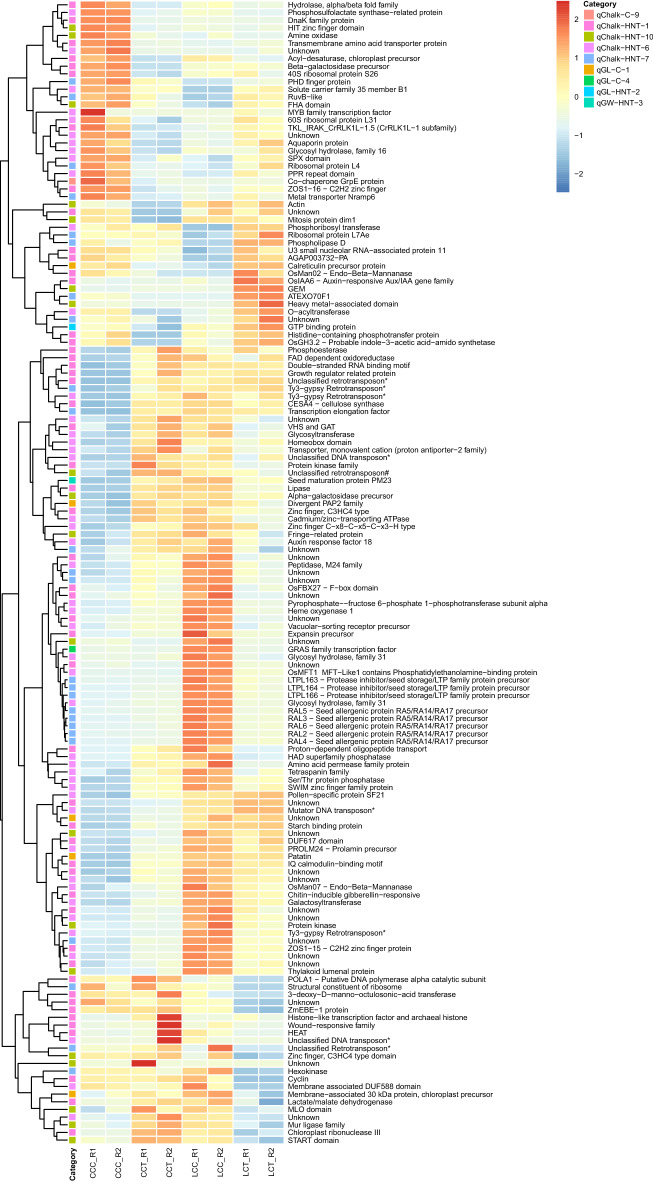
Heat maps showing the differential gene expression profiles and annotations of 149 differentially expressed genes (DEGs) at *log*_*2*_*fold change* ≥ 1 and *padj* < 0.05, which were assigned to their respective QTLs related to grain length, grain width, and percent chalkiness under high nighttime temperature (HNT) stress. CCC_R1 and CCC_R2 are two replicates for Cypress under control conditions, CCT_R1 and CCT_R2 are two replicates for Cypress under HNT stress conditions, LCC_R1 and LCC_R2 are two replicates for LaGrue under control conditions, and LCT_R1 and LCT_R2 are two replicates for LaGrue under HNT stress conditions.

To understand the effect of HNT stress in a genotype-dependent manner, differential gene expression analysis was performed on the genotype × environment (G × E) interaction of HNT stress treated and control replicates of the Cypress and LaGrue genotypes as described earlier. We aimed to identify candidate genes involved in GL, GW, and % chalk under HNT stress, by integrating QTL and expression QTL (eQTL) analyses of both parents. Of the 7117 genes, 149 genes were either up- or down-regulated in response to HNT stress at log_2_fold change ≥ 1 and *padj* < 0.05 as shown (Fig. 6). The genes showing significant differences for gene expression were assigned to their respective QTLs based on their genomic locations/co-ordinates. Of these 149 differentially expressed genes (DEGs), a subset of 35 genes (six for GL, one for GW, and 28 for % chalk) was identified based on the known function in starch biochemical pathways, stress-responsive pathways, and gene ontology (GO) based gene functions. The expression profiles and annotations of these genes are also shown (Supplementary Fig. S5). The subset comprises genes such as the Beta-galactosidase 4 (LOC_Os01g65460) which is downregulated in Cypress under HNT stress but highly expressed in LaGrue under HNT stress. Other examples of GO based genes included two loci with unknown gene functions, LOC_Os01g55160 and LOC_Os01g64330, that have been reported to be upregulated under endoplasmic reticulum (ER) stress during rice endosperm development and could cause aggregation of abnormal PB bodies that ultimately affect the grain quality^95^.

To identify the potential candidate genes, we followed a two-tier approach based on differential gene expression and allele mining to find SNPs that overlap with the 149 DEGs showing significant changes in expression under control and HNT conditions. We found 5,507 SNPs overlapped with the 149 DEGs ± 10 Kbp (14 SNPs or 0.25% of these were HISs that differentiate between the Cypress and LaGrue genotypes). This approach identified 11 candidate loci of interest (DEGs at log_2_FC ≥ 1 and *padj* < 0.05) and high impact SNP associations with these loci based on the position, predictive cause, and effect of the SNP (Table 4).

**Table 4:** Candidate genes identified using a two-tier approach of overlapping differentially expressed genes (*log2FC* ≥ 1 *and padj* < 0.05) and allele mining in the 51 Mbp region spanning the 15 QTLs.

Gene	Type	No. of HISs	Gene annotation
**LOC_Os06g25760**	**Protein_coding**	**15**	**Retrotransposon protein, putative, Ty3-gypsy subclass, expressed**
**LOC_Os06g42710**	**Protein_coding**	**3**	**Transposon protein, putative, Mutator sub-class, expressed**
LOC_Os01g60050	Protein_coding	2	HEAT, putative, expressed
**LOC_Os01g64330**	**Protein_coding**	**2**	**Retrotransposon protein, putative, unclassified, expressed**
LOC_Os07g16330	Protein_coding	2	Expressed protein
LOC_Os01g53560	Protein_coding	1	Phosphoesterase, putative, expressed
LOC_Os01g54300	Protein_coding	1	OsMan02—Endo-Beta-Mannanase, expressed
LOC_Os06g19980	Protein_coding	1	MYB family transcription factor, putative, expressed
LOC_Os06g45360	Protein_coding	1	Peptidase, M24 family protein, putative, expressed
LOC_Os07g12650	Protein_coding	1	Ribosomal protein L7Ae, putative, expressed
**LOC_Os07g16690**	**Protein_coding**	**1**	**Retrotransposon protein, putative, Ty3-gypsy subclass, expressed**

*HISs* high impact single nucleotide polymorphisms (SNPs).

Transposon derived protein coding genes are in bold.

## Discussion

HNT stress,one of the detrimental factors, negatively effects grain quality traits leading to poor grain quality in rice grains. Enhancing HNT stress tolerance with minimizing poor grain quality in US rice cultivars is one of the evolving targets in rice breeding. It has been determined that HNT stress (≥ 27 °C) during grain filing days in *japonica* rice triggers impairment of starch biosynthesis resulting into the increased chalkiness^29^. The increase in chalkiness alters the amylose content declining rice grain shape and size^33^. To improve the grain quality under HNT stress, it is extremely essential to identify favorable alleles linked with candidate genes related to grain quality traits using genome to phenome-assisted approaches. Here, we have carried out phenomics analysis of MY2 RIL population with both parents “Cypress and LaGrue” for grain quality traits under HNT stress and control conditions (Fig. 1), which allowed us to map and deeply characterize the genomic region linked with grain quality traits under HNT stress.

The broad range of phenotypic variation for grain quality traits under HNT stress and control conditions in the population indicates that GL, GW, and % chalk come up with quantitative inheritance that would be useful for QTL mapping. Our findings revealed that the parent Cypress showed least reduction in GL, GW, and smaller increase in % chalk while other parent LaGrue exhibited significantly higher reduction in GL, GW, and greater increase in % chalk under HNT stress compared to control conditions. So, the significant reduction in GL and GW is related to decline in carbohydrate availability due to high nighttime respiration or reduced carbon assimilates supply under HNT stress in rice^96^. Thus, the activity of several enzymes involved in biosynthesis and deposition of starch in the endosperm, negatively impacts grain dimensions and grain appearance in rice^96^ and other cereal crops^97^. The investigation of genetic variation derived from the both parents in the MY2 RIL populations, under HNT stress conditions, showed the highest genetic variation in % chalk followed by GW and GL (Fig. 2A–C, Table 1). Our results suggest that there was transgressive variation in the RIL populations exhibiting larger phenotypic values of each quality trait compared to both parents under prolonged HNT stress. This indicates that positive and negative alleles or loci with epistasis or additive effects, derived from both parents, can function together to substantially affect the GL, GW, and % chalk^91^. Several studies have shown that grain appearance is primarily determined by grain shape & size, a combination of GL, GW, and grain thickness (GT)^9,18^. During grain filling stage (R6 stage), air temperature in the environment plays an important role in generating unexpected variations in rice grain quality^11^. High broad sense heritability and relationship between grain quality traits under control and HNT stress conditions indicate the strong trait stability in different environments and strong genetic control directing the selection of genotypes-based GL, GW, and % chalk for HNT stress tolerance, which can be used in rice breeding.

The physical appearance traits (GL, GW, and chalkiness) determining grain quality in rice are important to rice growers and consumers. Most of the rain appearance traits such as GL, GW, and length and width ratio are heritable and can be selected through conventional breeding approaches; however, significant improvement in grain architecture such as chalkiness is difficult to achieve^98^. Currently, low-price sequencing of rice genomes and availability of advanced genomics tools and technologies have re-imagined rice breeding for mapping the QTLs/genes related to grain yield and quality. The grain quality traits including grain length, width, ratio of length & width, and chalkiness are the polygenic quantitative traits^34^. To date, more than 200 QTLs for GL and GW^36^ and more than 100 QTLs for chalkiness components have been identified in *Indica* and *indica-japonica* background of rice^34,40,42,43^. However, until now, this is first study focusing on mapping and characterization of QTLs for quality traits in the *TRJ* background RIL population derived two elite *TRJ* US rice cultivars “Cypress and LaGrue” under HNT stress. In the QTL mapping analysis, the additive effects suggest that the favorable alleles with negative effect were contributed by Cypress and favorable alleles with positive effect were come from LaGrue (Table 2). A QTL with more than 20% PVE was considered as a major effect QTL. Thus, among 9 additive effect QTLs for GL, GW, and %chalk under HNT stress, 4 QTLs related to % chalk are major effect-QTLs showing more than 20% phenotypic variance and these QTLs were mapped on chr1, ch6, ch7, and chr10. The higher phenotypic variation explained for most of the studied %chalk revealed major genes/QTLs to be responsible. To analyze the QTLxQTL interaction, surprisingly, there was no QTLXQTL interaction for GL and GW under HNT stress. However, for % chalk, locus on chr1 showed significant epistatic interaction with loci on chr2, chr4, chr5, ch6, chr7, chr8, chr10, chr11, and ch12 with PVE range from 0.62 to 2.37% and epistatic effect range from 0.50 to 2.79%. So, the epistatic interactions for % chalk in the genome suggest that the genomic region on chr1 plays an important role in controlling % chalk in rice grains.

Based on the physical positions of the SNP makers, identified QTLs related to GL, GW, and % chalk under control and HNT stress were investigated for co-localization with the previously reported QTLs for GL, GW, and chalkiness mapped in *indica-japonica* background. To validate the strength of the identified 15 QTLs for GL, GW, and % chalk under control and HNT stress conditions in the present study, 29 major effects QTLs related to grain quality traits (GL, GW, ratio of length and width, chalkiness components) reported in 15 independent publicly available studies in last two decades were analyzed^21,46,51,55,62,85–94^. Among 15 QTLs, 12 QTLs were coincided with the genomic regions of these previously reported 29 QTLs (Table 2, Fig. 5, Supplementary Fig. S4). These 12 QTLs for GL, GW, and % chalk in rice genome showed the strongest evidence of co-localization with other well-characterized and major effects previously reported QTLs, which suggest that these QTLs are consistently stable in the rice genome. The genomic regions of these QTLs bear useful gene targets to understand the HNT stress tolerance mechanism. Our co-localization analysis revealed that this information can be implemented in rice breeding using these SNPs for marker-assisted selection and introgression of the co-localized genomic regions with these QTLs of grain quality traits into other elite US rice cultivars to develop new rice cultivars. In addition, a follow-up search for these favorable alleles that we found in 83 rice accessions of the JDP will facilitate the selection of the trait phenotypes for other novel elite alleles.

To characterize the 51 Mbp of genomic region comprising15 QTLs for grain quality traits in the present study, a set of 83 rice accessions of the JDP was used to discover new favorable alleles using existing natural genetic variation. Among 868,823 polymorphic alleles, 131,269 alleles representing 15% of the total alleles are newly discovered alleles showing novel genomic variation between both parents, Cypress and LaGrue. Based on the SNP type, region, functional class, position, proximity to the gene or gene features, 6160 newly discovered SNPs/alleles (out of 868,823 total) are high impact SNPs (HISs), of which 865 HISs representing 14% of the total HISs have shown novel genetic variation between both parents. The HISs identified in this study are possible candidates for their use as functional markers to differentiate the two US cultivars, and thus find useful in genomics-assisted breeding and introgression studies. The SNPs that are unable to differentiate between Cypress and LaGrue are also useful in the sense that they indicate the presence of natural genetic variation for GL, GW, and % chalk in the *japonica* accessions of the JDP. The detailed summary of these SNPs with the genomic region of 15 QTLs showing highly dense distribution of SNPs/alleles in 83 rice genotypes of the JDP is shown in Fig. 5 and Supplementary Table S3. These observations emphasize the power of this analysis as there was a 2600-fold increase in SNP density over the initial set of markers that were used to identify the QTLs thus providing higher marker saturation (initial marker density = one marker every 155 Kb). Upon validation, these SNPs will provide a valuable resource for the future genomics-assisted breeding efforts for the traits of interest such as grain yield and grain quality.

In the transcriptome analysis, a total of 7117 genes were identified in the 51Mbp region comprising the 15 QTLs using IRGSP 1.0 genomic feature file (GFF) as a reference. Out of these, we identified 495 genes there either up- or down-regulated in response to HNT stress and showed significant variation for gene expression under control and HNT stress conditions in a genotype-dependent manner (Fig. 6). Narrowing down further, a subset of 35 genes associated with GL, GW, and % chalk under HNT stress were selected based on their known functions in starch biochemical pathways, stress-responsive pathways, and gene ontology (GO) (Supplementary Fig. S5). Two genes, LOC_Os01g55160 and LOC_Os_01g64330 with unknown functions, identified in this study, have previously been shown to be upregulated under endoplasmic reticulum (ER)R stress during endosperm development in rice, which could be responsible for aggregation of abnormal protein bodies overall affecting the rice gran quality^94^. Moreover, plant beta-galactosidases (B-gals) have been well characterized and are involved in degradation of structural pectin and xyloglucans in plant cell walls^98^. The observed expression pattern of the *B-gals 4* gene (LOC_Os01g65460) in this study is downregulated in Cypress HNT under stress; however, highly expressed in LaGrue under HNT stress, therefore alludes to starch degradation in LaGrue under HNT stress as compared to Cypress.

Other genes identified in this study are thought to have potentially relevant roles such as in starch accumulation, endosperm formation &development, stress response, grain size, grain quality, chalkiness etc., some of which are described below in detail^99–104^. Five potential candidate genes identified in the genomic region of QTL, *qChalk7HNT*, belong to α-amylase/trypsin inhibitors upregulated in Cypress under HNT stress conditions. In addition, upregulation of OsDGAT1-2, *LOC_Os06g36800*, in LaGrue under HNT stress could be a cause of increased chalkiness because of accumulation of triacylglycerol (TAG) and lipid bodies, thereby hindering proper starch accumulation under HNT stress^100^. The cyclin protein, LOC_Os01g59120, is 7× upregulated in Cypress under HNT stress vs LaGrue under HNT stress and plays a critical role in endosperm formation and maintenance of embryo development in rice^101^. The upregulation of LOC_Os06g38790, an orthologue gene (ZmEBE-1) of maize, in rice may play a role under HNT stress conditions in Cypress under HNT stress vs LaGrue under HNT stress in early development of specialized domains of the endosperm^101^. OsHAP3//nuclear factor-YB (NF-YB)/CCAAT binding factor-A (CBF-A) in rice regulates chloroplast plastid biogenesis^103^. OsLMS, *LOC_Os01g63820*, shares amino-acid similarity to the Arabidopsis FIERY2/CPL1 gene, known to control many plant stress responses and development^104^. FERONIA-like receptor (*FLR1*) family members’ effect grain size & quality and stress tolerance, and *FLR2* and *FLR8* negatively regulate grain size^105^. Downregulation of *FLR2* gene in Cypress under HNT stress suggests maintaining grain filling compared to LaGrue under HNT stress condition. The cumulative slight perturbation in the expression of multiple genes at multiple loci under HNT stress could cause a QTL for a particular trait to be additive and epistatic. Similarly, the upregulation of a gene in non-photosynthetic tissue (endosperm) in Cypress under HNT stress vs LaGrue under HNT stress could have a potential role in amyloplastic formation.

To find the candidate genes for grain quality in US *japonica* rice, we followed a two-tier approach using differential gene expression and newly discovered favorable alleles/SNPs overlapped with the 149 DEGs exhibiting significant changes in the expression under control and HNT stress identified 11 candidate genes with high impact SNP associations (Table 4). Of particular interest is the HEAT protein (LOC_Os01g60050), a microtubule organization protein that is essential for cortical microtubule organization in plant cells and is speculated to play an important role during cellurization^106^. This gene is stress responsive and is 12× upregulated in Cypress (HNT tolerant parent) as compared to LaGrue (HNT sensitive parent) under HNT stress. It is also worth noting that four out of 11 loci are transposon derived protein-coding genes that are marred with HISs and show differential gene expression under HNT stress. For instance, the loci *LOC_Os06g25760-a* retrotransposon belonging to the Ty3-gypsy subclass contains 15 HISs. Such rapid rate of mutations accumulating SNPs at a higher rate in transposons as compared to genes is a meaningful observation from an evolutionary perspective of genome structure and function. Additionally, due to their presence in the transcriptome, these transposons are presumably active in the genome and are differentially expressed in a stress responsive manner. This makes them candidates of choice to study transposon-mediated regulation of genes involved in HNT stress response.

The power of allele mining using the top-down approach to integrate the genomic and transcriptomic datasets has its takeaway. Of the possible 6.1 million SNPs distributed across the 12 chromosomes or 868,823 SNPs distributed across the 15 QTLs on nine chromosomes, we narrowed down to a subset of 30 High Impact SNPs for further testing (Table 4). Additionally, from a total of ~ 48 K genes in the rice genome, we shortlisted a potential set of 11 protein coding genes including transposons for validation and characterization (Table 4). These genes are important because i. They reside in the QTL regions of interest, ii. They are differentially expressed between the parental genotypes “Cypress” and “LaGrue” in a stress responsive manner, and iii. They harbor the HISs and could be functionally relevant. Besides increasing the marker saturation of the initial set of 1178 SNP markers by 2600-fold, we identified a set of polymorphic markers that distinguish between two parents, Cypress and LaGrue, that can be put to future use for introgression or other breeding strategies.

## Conclusions

This is the first integrated study to screen the MY2 RIL population (185 lines) derived from two US rice cultivars, “Cypress and LaGrue”, for grain quality traits such as GL, GW, and % chalk at booting stage (R2: reproductive stage) under HNT stress, map & characterize the QTLs regions by mining for novel alleles and identify the candidate genes for grain quality under HNT stress. Phenotypic screens indicated that the MY2 RIL population with both parents (US rice cultivars) showed a wide range of variation for GL, GW, and % chalk under HNT stress compared to control conditions and the RIL population exhibited strong trait correlation and high broad sense heritability for grain quality traits under HNT stress. Thus, the phenotyping insights suggest that the MY2 RIL population exhibited transgressive segregation that would be useful for QTLs mapping and their characterization to find the candidate genes for GL, GW, and %chalk under HNT stress. Co-localization of 15 QTLs with the previously reported 29 major effects-QTLs for GL, GW, and % chalk validates the robustness of the favorable alleles linked with the QTLs and strengthens the QTL analysis in this study. Further characterization of the QTLs through allele mining and identification of genes that showed genotype dependent differential expression under HNT stress and control conditions led us to identify 11 candidate genes with high impact SNP associations based on the position, function, and predictive cause/effect of the SNPs. Therefore, all the findings from this study are noteworthy and the approach used demonstrates the power of integration of phenotypic, genomic, and transcriptomic data for gaining insights into genetic variations to understand the mechanisms responsible for heat stress tolerance that contribute to the grain quality in rice. These insights will be useful for developing allele-specific markers for genotyping US (*japonica* background) rice germplasms/elite breeding lines, optimizing genomic predictions for breeding program, establishing SNPs-assisted breeding strategies to breed superior varieties with improved grain quality and yield.

## Supplementary Information

Supplementary Information.

## Data Availability

The SNPs and gene expression datasets generated and analyzed during the current study are available in European Variation Archive (PRJEB57338) and Gene Expression Omnibus (GSE220996), respectively.
