# Supplementary material for: Molecular mapping and characterization of QTLs for grain quality traits in a RIL population of US rice under high nighttime temperature stress
Source: Sci Rep. 2023 Mar 25;13:4880. doi: 10.1038/s41598-023-31399-w (PMC10039871; doi:10.1038/s41598-023-31399-w)
Supplement: Supplementary file 1 — Supplementary Information. [file 41598_2023_31399_MOESM1_ESM.pdf]

Molecular mapping and characterization of QTLs for grain quality traits in a RIL population of US rice under high nighttime temperature stress

Anuj Kumar[1], Julie Thomas[1†], Navdeep Gill[2†], Yheni Dwiningsih[1], Charles Ruiz[1], Adam Famoso[2], Andy Pereira[1*]

[1]Departemnt of Crop, Soil, & Environmental Sciences, University of Arkansas, Fayetteville, AR 72701, USA
[2]Department of Biological Sciences, Nova Southeastern University, Fort Lauderdale, FL 33314, USA
[3]H. Rouse Caffey Rice Research Station, Louisiana State University Agricultural Center, Rayne, LA 70578, USA

[†] equally contributed authors

*Email: apereira@uark.edu

S.No	GSOR #[a]	Accession Name	Ancestry	Country
1	GOSR301408	KAYBONNET	Tropical Japonica	United States
2	GSOR301299	Cypress	Tropical Japonica	United States
3	GSOR301300	LaGrue	Tropical Japonica	United States
4	GSOR301418	Bengal	Tropical Japonica	United States
5	GSOR310007	Karang Serang	Tropical Japonica	Indonesia
6	GSOR310020	E B Gopher	Tropical Japonica-Temperate Japonica	United States
7	GSOR310023	RD 218	Tropical Japonica-Temperate Japonica-Aromatic	Dominican Republic
8	GSOR310045	LEAH	Tropical Japonica	United States
9	GSOR310052	Quinimpol	Tropical Japonica	Philippines
10	GSOR310080	TAICHU MOCHI 59	Tropical Japonica	Taiwan
11	GSOR310087	WC 2811	Tropical Japonica	Micronesia
12	GSOR310102	Criollo Chivacoa 2	Tropical Japonica	Venezuela
13	GSOR310111	Bombilla	Temperate Japonica	Spain
14	GSOR310131	Secano do Brazil	Tropical Japonica	El Salvador
15	GSOR310144	British Honduras Creole	Tropical Japonica	Belize
16	GSOR310156	Sel. No. 388	Tropical Japonica-Temperate Japonica	Uruguay
17	GSOR310161	SHIMIZU MOCHI	Temperate Japonica	Japan
18	GSOR310204	Italica Carolina	Temperate Japonica	Poland
19	GSOR310210	KRASNODARSKIJ 424	Temperate Japonica	Russian Federation
20	GSOR310211	Pergonil 15	Temperate Japonica	Portugal
21	GSOR310226	NORIN 11	Temperate Japonica	Japan
22	GSOR310238	R 75	Temperate Japonica	Senegal
23	GSOR310241	UZ ROSZ M38	Temperate Japonica	Uzbekistan
24	GSOR310301	H57-3-1	Temperate Japonica	Argentina
25	GSOR310338	Khao Luang	Tropical Japonica-Temperate Japonica-Aromatic	Laos
26	GSOR310345	J.P. 5	Temperate Japonica	Australia
27	GSOR310348	C 8429	Tropical Japonica	Papua New Guinea
28	GSOR310354	Padi Pohon Batu	Tropical Japonica	Malaysia
29	GSOR310397	Chacareiro Uruguay	Temperate Japonica	Uruguay
30	GSOR310428	Sipirasikkam	Tropical Japonica	Indonesia
31	GSOR310510	BLUE STICK	Temperate Japonica	Fiji
32	GSOR310588	Onu B	Tropical Japonica	Zaire
33	GSOR310645	Moroberekan	Tropical Japonica	Guinea
34	GSOR310670	KUBANETS 508	Temprate Japonica	Russian Federation
35	GSOR310747	BHIM DHAN	Tropical Japonica-Temperate Japonica-Aromatic	Nepal
36	GSOR310767	HB-6-2	Temperate Japonica	Hungary
37	GSOR310777	WC 3532	Tropical Japonica	Peru
38	GSOR310779	GPNO 1106	Tropical Japonica	Guatemala
39	GSOR310799	Ragasu	Tropical Japonica-Temeprate Japonica	Taiwan
40	GSOR310801	Tobura	Temperate Japonica	Taiwan
41	GSOR310802	Tamanishiki	Temperate Japonica	Japan
42	GSOR310809	Yong Chal Byo	Temperate Japonica	Korea, South
43	GSOR310814	Grassy	Tropical Japonica	Haiti
44	GSOR310836	GPNO 5055	Tropical Japonica	United States
45	GSOR310861	Niwahutaw Mochi	Temperate Japonica	Japan
46	GSOR310879	6360	Temperate Japonica	Turkey
47	GSOR310883	Somewake	Temperate Japonica	Japan

48	GSOR310887	Buphopa	Tropical Japonica-Temperate Japonica-Aromatic	Myanmar
49	GSOR310906	Ardito	Temperate Japonica	Italy
50	GSOR310950	NANTON NO. 131	Tropical Japonica-Temperate Japonica-Aromatic	Taiwan
51	GSOR310958	2	Aromatic	Afghanistan
52	GSOR310965	Vary Tarva Osla	Temperate Japonica	Portugal
53	GSOR310984	CSORNUJ	Temperate Japonica	Hungary
54	GSOR310997	LUSITANO	Temperate Japonica	Portugal
55	GSOR310998	WC 4443	Temperate Japonica	Bolivia
56	GSOR311074	Mitak	Tropical Japonica	Indonesia
57	GSOR311078	Gazan	Temperate Japonica	Afghanistan
58	GSOR311151	TD 70	Temperate Japonica	Thailand
59	GSOR311185	Bombon	Temperate Japonica	Spain
60	GSOR311206	79	Aromatic	Guyana
61	GSOR311258	Botika S/R	Tropical Japonica	Zaire
62	GSOR311327	Gasym Hany	Aromatic	Azerbaijan
63	GSOR311383	DARMALI	Tropical Japonica-Temprate Japonica-Aromatic	Nepal
64	GSOR311385	KAUKKYI ANI	Tropical Japonica	Myanmar
65	GSOR311393	Celiaj	Temperate Japonica	Azerbaijan
66	GSOR311532	Egyptian Wild Type	Temperate Japonica	Turkey
67	GSOR311537	A 5	Temperate Japonica	Japan
68	GSOR311600	Jyanak	Tropical Japonica-Temprate Japonica-Aromatic	Bhutan
69	GSOR311620	Romeno	Temperate Japonica	Portugal
70	GSOR311642	Tia Bura	Tropical Japonica	Indonesia
71	GSOR311643	Padi Tarab Arab	Tropical Japonica	Malaysia
72	GSOR311677	Karabaschak	Temperate Japonica	Bulgaria
73	GSOR311685	WIR 911	Temperate Japonica	Russian Federation
74	GSOR311710	Lua Chua Chan	Tropical Japonica	Vietnam
75	GSOR311735	Simpor	Tropical Japonica	Brunei
76	GSOR311736	Coppocina	Tropical Japonica	Bulgaria
77	GSOR311769	Pakkali	Aromatic	Philippines
78	GSOR311779	WC 10253	Tropical Japonica	United States
79	GSOR311787	KRASNODARSKIJ 3352	Temprate Japonica	Russian Federation
80	GSOR311788	EMBRAPA 1200	Tropical Japonica	Brazil
81	GSOR311790	WAB462-10-3-1	Tropical Japonica	Cote D'Ivoire
82	IRAT177	IRAT177	Tropical Japonica	United States
83	M204	M204	Temperate Japonica	United States

Supplementary Table S1. List of 83 rice accessions of the *japonica* diversity panel (JDP) was used for the characterization of 15 QTLs region (51mb) of the genome for natural genetic variation in this study. [a]GSOR #: Genetic Stock Oryza Number given by the USDA.

Trait	Treat[a]	1st Chr [b]	Pos.1 (bp)[c]	2nd Chr[d]	Pos.2 (bp)[e]	LOD[f]	PVE (%)[g]	Add-by-Add[h]
%Chalk	Control	chr1	30,498,826-38,418,739	chr3	2,876,421-3,385,050	4.4828	2.3763	1.101
				chr4	30,145,846-33,144,611	4.6293	2.3749	-1.055
%Chalk	HNT	chr1	30,498,826-38,418,739	chr2	21,329,057-22,020,329	5.1149	0.7687	1.318
				chr3	7,344,766-31,565,725	4.7216	0.7669	-6.077
				chr4	16,285,717-16,328,027	5.991	0.7844	-1.497
				chr5	6,607,616-14,632,867	4.9179	0.7912	-4.981
				chr6	353,198-9,977,282	5.6878	0.8137	-6.062
				chr7	18,622,810-25,944,766	4.3781	0.6214	-4.641
				chr8	22,618,303-24,452,287	4.7721	0.7598	1.017
				chr10	13,710,808-16,820,816	4.1498	0.6806	-0.12
				chr11	17,586,119-27,820,001	4.2733	0.7341	-0.451
				chr12	22,740,633-25,670,526	4.8135	0.6337	-4.499
%Chalk	Control	chr2	17,042,797-18,971,451	chr11	16,406,515-17,355,355	4.1789	0.5014	-0.443
%Chalk	HNT	chr2	19,270,353-21,084,606	chr5	6,607,616-14,632,867	5.0285	0.5612	5.015
		chr2	17,042,797-18,971,451	chr7	770,090-15,703,246	5.3745	0.8228	1.451
		chr2	5,708,391-6,084,918	chr10	17,908,351-21,817,967	4.1413	0.5038	5.391
GL	Control	chr3	5,889,025-27,134,184	chr8	17,621,423-21,869,284	4.1052	2.7964	0.134
%Chalk	HNT	chr3	5,889,025-27,134,184	chr7	770,090-15,703,246	4.2172	0.644	4.562
		chr3	29,561,125-31,627,459	chr9	4,052,650-6,667,280	4.2104	0.7108	5.477
%Chalk	Control	chr4	27,702,782-29,289,031	chr9	4,052,650-6,667,280	5.0287	2.3679	1.072
%Chalk	HNT	chr4	29104579-31,576,593	chr5	2,2634,432-23,972,013	4.6491	0.5921	5.349
		chr4	15,979,347-19,567,187	chr7	770,090-15,703,246	6.1343	0.8744	-2.203
%Chalk	Control	chr5	22,634,432-23,972,013	chr9	4,052,650-6,667,280	4.1468	2.2978	-1.015
%Chalk	HNT	chr5	6,607,616-14,632,867	chr7	770,090-15,703,246	5.8139	1.0094	-8.234
%Chalk	HNT	chr6	1,768,006-4,644,998	chr7	18,622,810-25,944,766	4.7162	0.6527	4.994
		chr6	28,061,782-29,142,501	chr9	4,052,650-6,667,280	4.2881	0.7856	3.267
GL	Control	chr7	2,721,936-4,738,030	chr8	6,262,287-9,200,680	4.0088	2.6878	0.109
%Chalk	HNT	chr7	770,090-15,703,246	chr9	4,052,650-6,667,280	4.078	0.76	3.357
			770,090-15,703,246	chr11	3,390,991-3,956,996	4.0412	0.8101	1.388
			770,090-15,703,246	12	20,778,689-22,774,194	4.2714	0.7555	-3.642
GW	Control	chr9	17,736,891-18,819,334	10	11,929,159-13,428,212	4.4975	1.8468	0.036

Supplementary Table S2. Epistatic QTLs identified for grain quality traits in the MY2 RIL population derived from cross of two US rice cultivars Cypress and LaGrue using ICIM-EPI method implemented in QTL IciMapping. [a]Trt: treatment conditions (Control and high nighttime temperature-HNT), [b]1st Chr: rice chromosome on which first QTL involved in epistasis, [c]Pos.1(bp): position in base pair of flanking marker of first QTL in epistasis on specific chromosome, [d]2nd Chr: rice chromosome on which second QTL involved in epistasis, [e]Pos.2(bp0: position in base pair of flanking marker of second QTL in epistasis on specific chromosome, [g]LOD: logarithm of the odds peak/score was set >4.0 as threshold for detecting significant epistatic interaction between two QTLs, [g]PVE(%): total phenotypic variance explained by the QTL in percentage (%), [h]Add-by-Add: epistatic effect between two QTLs.

Rice genotype	All SNPs		High Impact SNPs (HISs) only	
	SNP count*	SNP density**	SNP count*	SNP density**
83 genotypes of the JDP	868,823	59	6,160	8,306
Cypress-LaGrue (Parents)	131,629	389	865	59,151

Supplementary Table S3. Summary of SNP counts and SNP density identified by allele mining in the 83 genotypes of the japonica diversity panel (JDP) and SNPs that differentiate the two parental genotypes, “Cypress and LaGrue”. *Total Polymorphic site counts in the 51 Mbp region spanning the 15QTLs. **SNP density is 1 SNP every bp calculated as cumulative QTL length of 51,165,717bp/number of SNPs

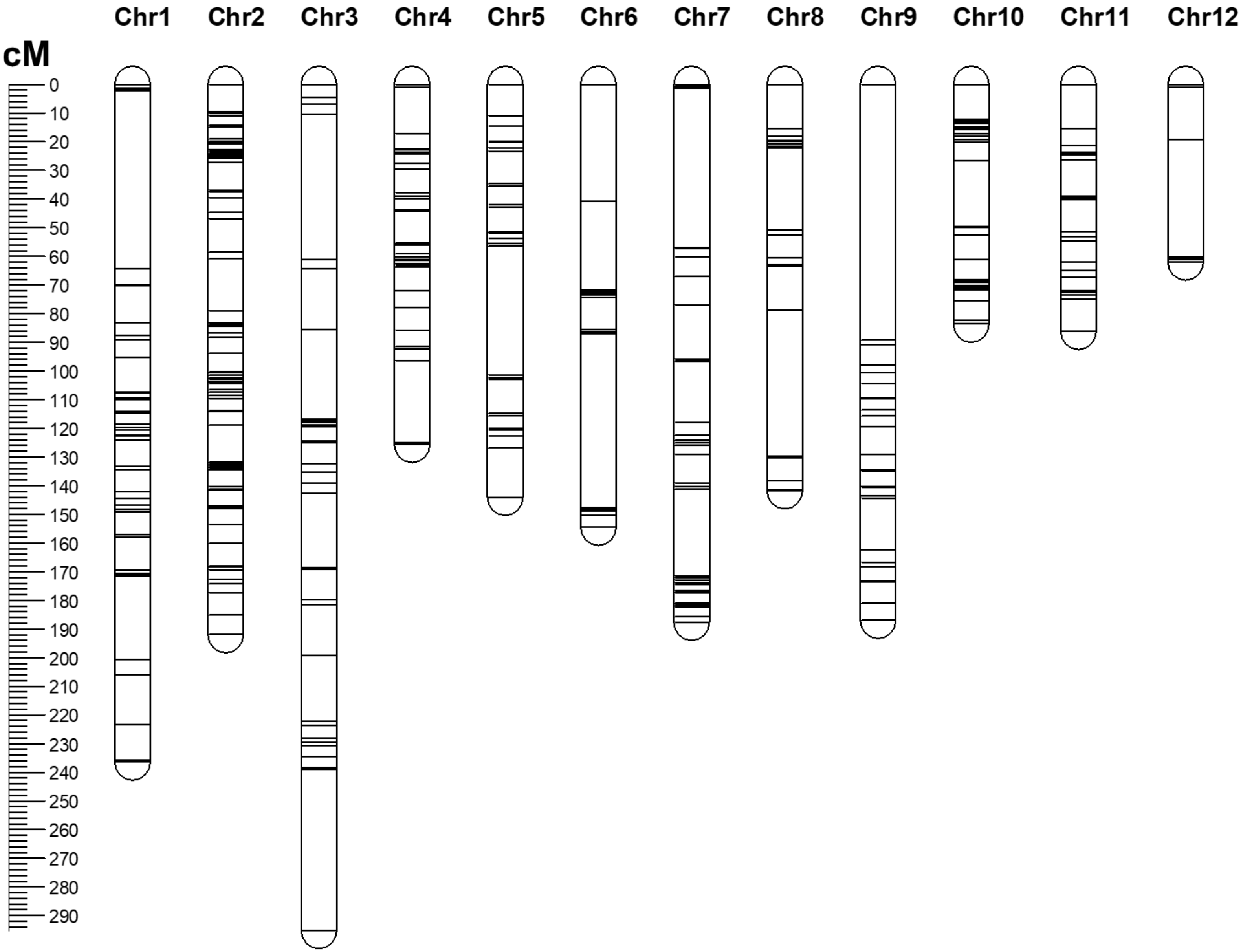

Supplementary Figure S1. The genetic linkage map constructed using 1178 SNP markers showing a total length of 1897.87cM with an average marker density of 1.6 markers per cM in the rice genome.

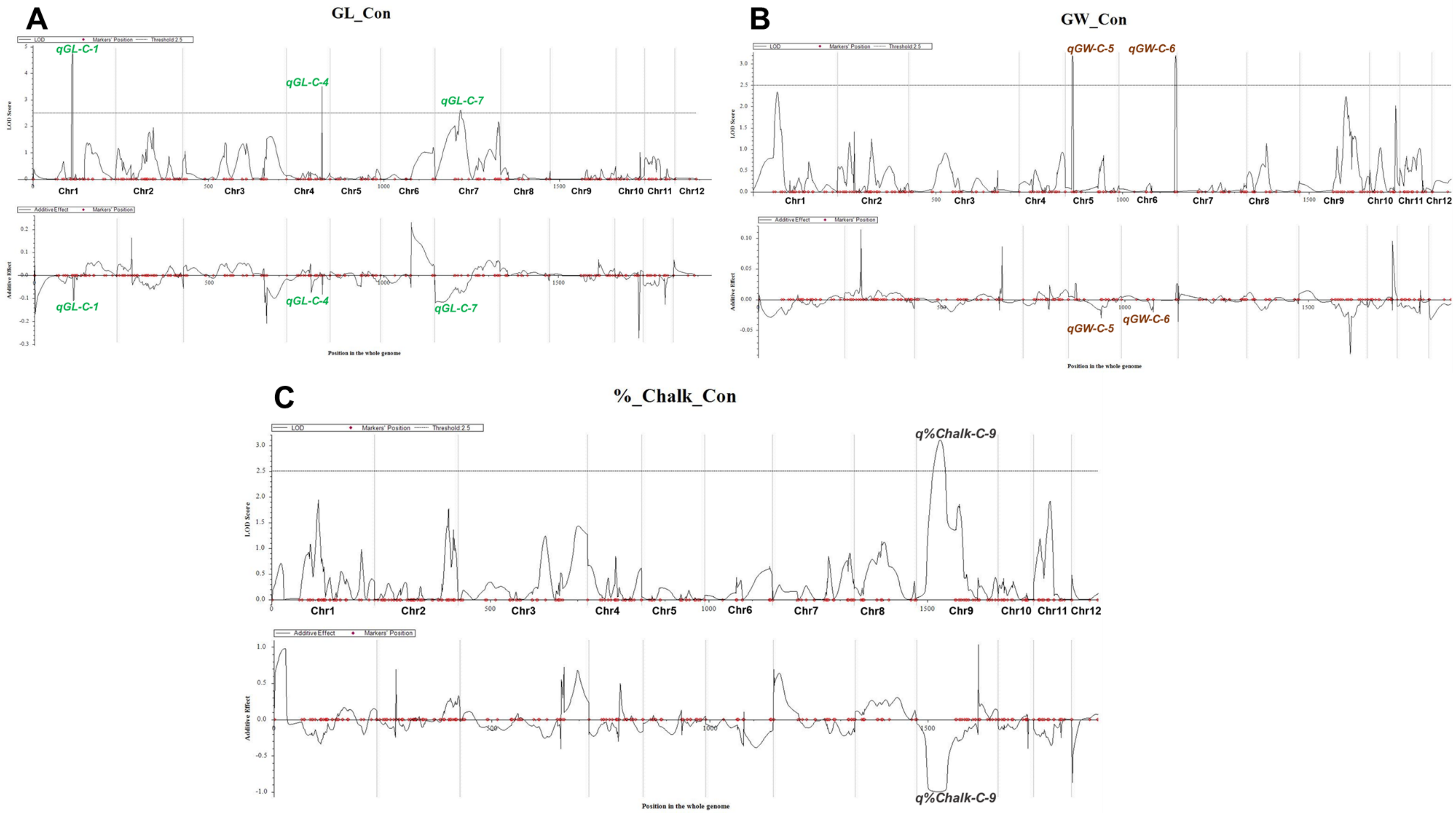

Supplementary Figure S2. Quantitative trait loci (QTLs) mapping, for grain quality traits under control condition, by ICIM-ADD mapping implemented by IciMapping 4.0. The rice genome was scanned for 6 QTLs under control condition in the MY2 RIL population derived from two US rice cultivars, "Cypress and LaGrue". A) Three QTLs (on the top) with their additive effects (in the bottom) for grain length (GL). B) Two QTLs (on the top) with their additive effects (in the bottom) for grain width (GW). C) One QTL (on the top) with its additive effect (in the bottom) for percent chalkiness). The dotted horizontal lines indicate the LOD threshold to declare the significant QTL.

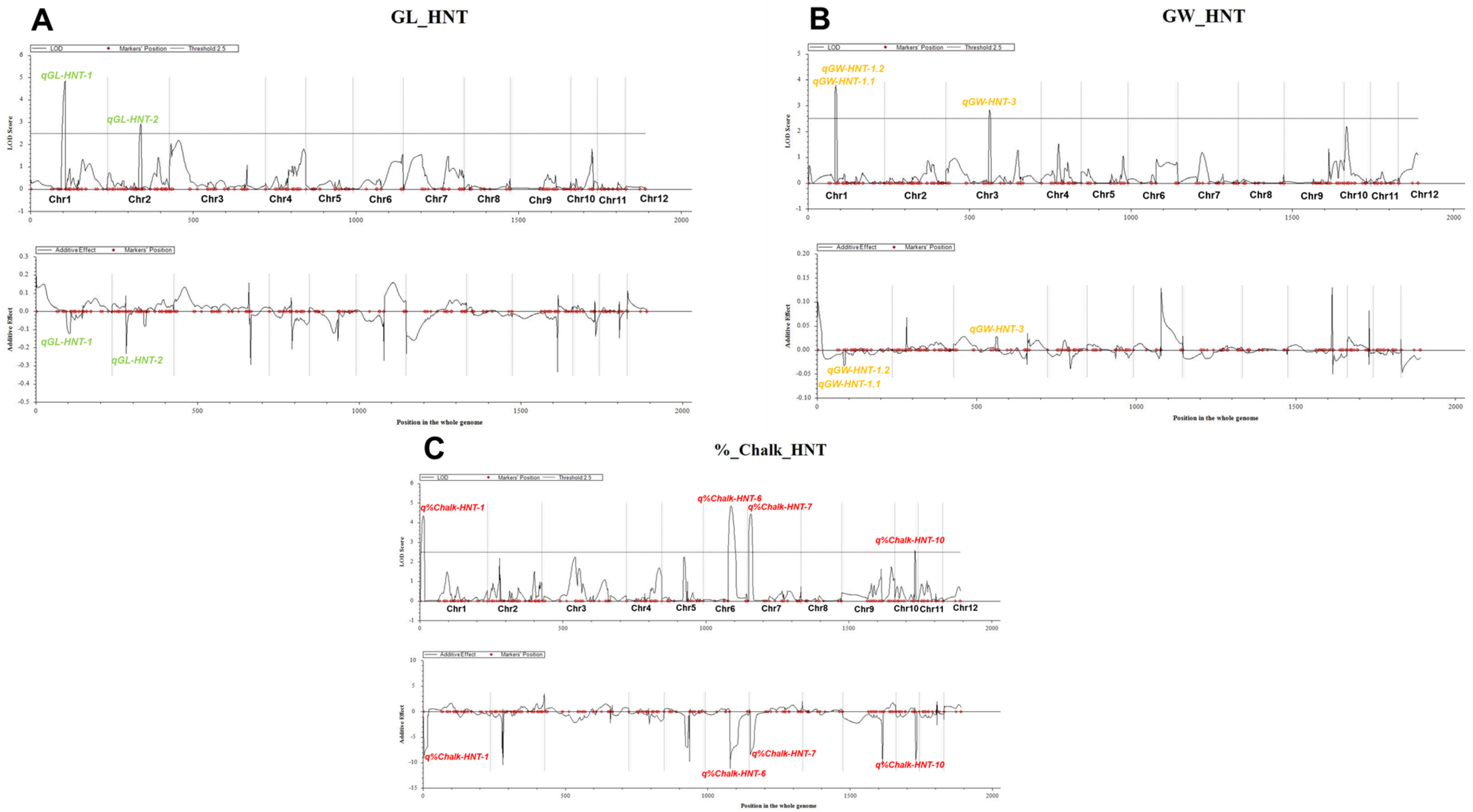

Supplementary Figure S3. Supplementary Figure S2. Quantitative trait loci (QTLs) mapping, for grain quality traits under high nighttime temperature (HNT) stress, by ICIM-ADD mapping implemented by IciMapping 4.0. The rice genome was scanned for 9 QTLs under HNT stress in the MY2 RIL population derived from two US rice cultivars, “Cypress and LaGrue”. A) Two QTLs (on the top) with their additive effects (in the bottom) for grain length (GL). B) Three QTLs (on the top) with their additive effects (in the bottom) for grain width (GW). C) Four QTLs (on the top) with their additive effect (in the bottom) for percent chalkiness). The dotted horizontal lines indicate the LOD threshold to declare the significant QTL.

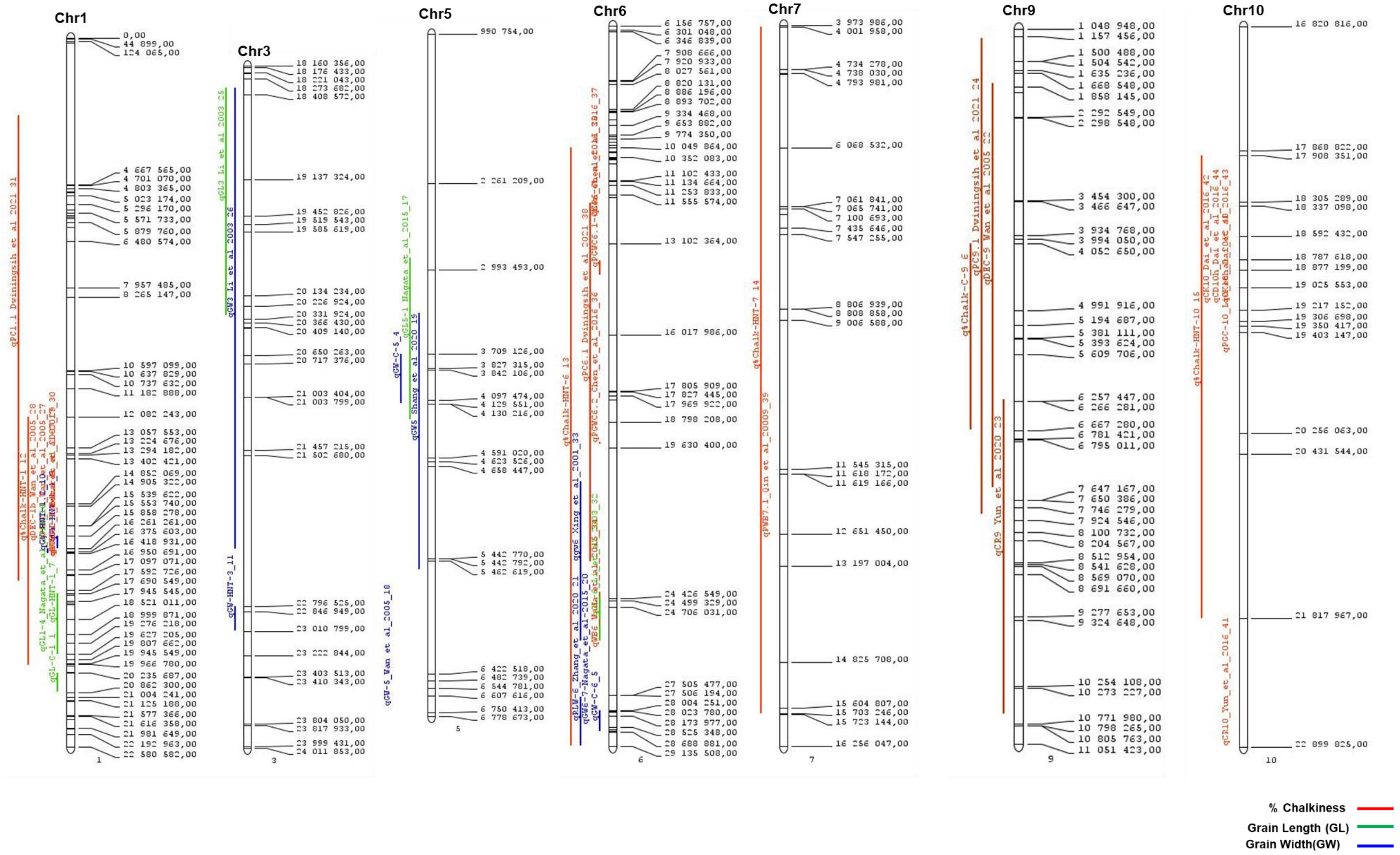

Supplementary Figure S4. Comparative physical map of the 15 quantitative trait loci (QTLs) for grain length, grain width, and percent chalkiness identified in this study under control and high nighttime temperature (HNT) stress conditions. Among 15 QTLs, 12 QTLs were co-localized with genomic regions of previously reported 29 major effects-QTLs in 15 independent studies in *japonica* rice background around the world. On each chromosome, the genomic positions (in bp) of the markers are shown on the right-hand side, while 12 QTLs along with previously reported QTLs and their annotations highlighted on the left-hand side. Each QTL for individual grain quality trait is represented by an independent color as highlighted.

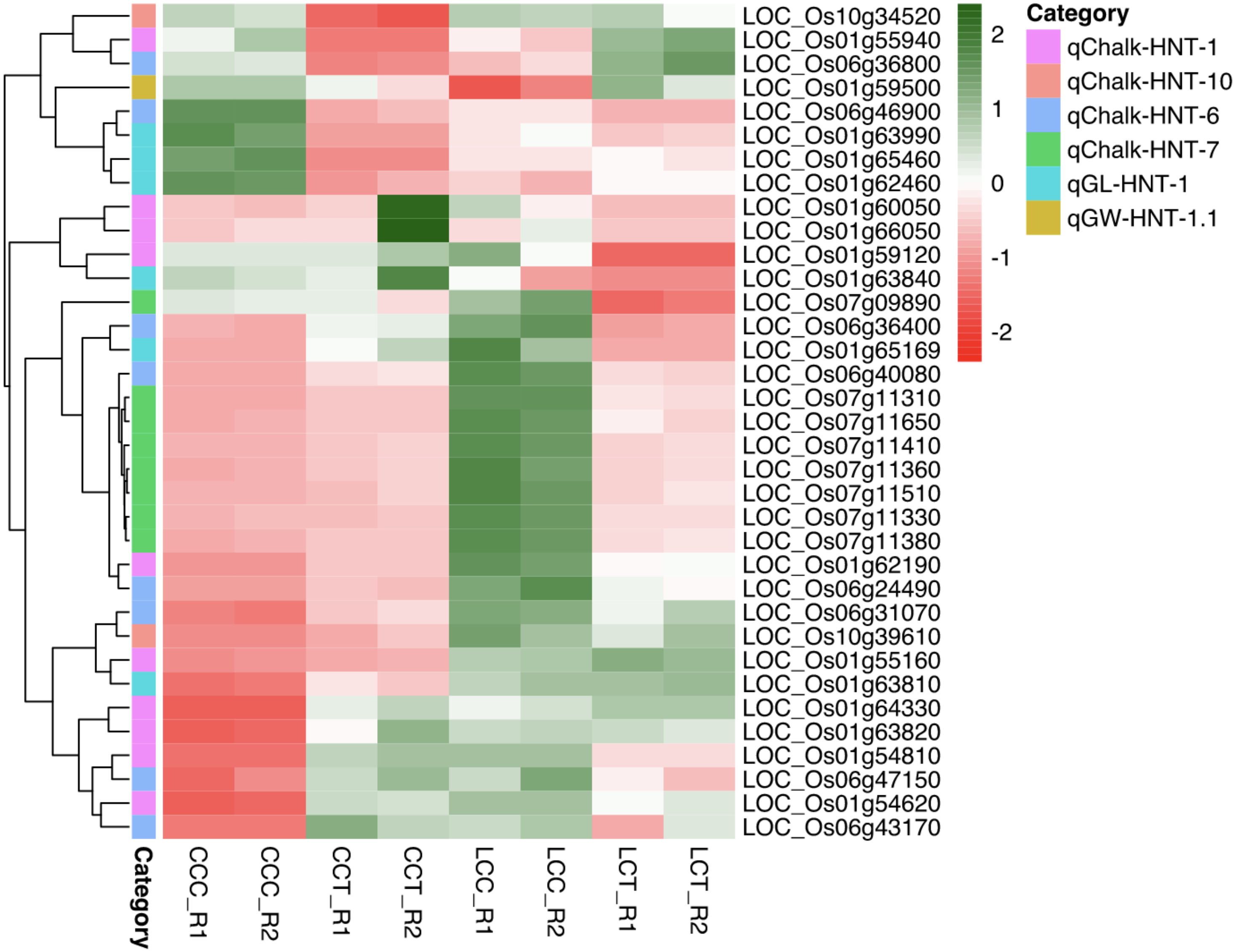

Supplementary Figure S5. Heat maps showing the differential gene expression profiles and annotations of a subset of 35 differentially expressed genes (DEGs) at *log2FC>=1* and *padj<0.05*, which were assigned to their respective QTLs related to grain length, grain width, and percent chalkiness under high nighttime temperature (HNT) stress. CCC_R1 and CCC_R2 are two replicates for Cypress under control conditions. CCT_R1 and CCT_R2 are two replicates for Cypress under HNT conditions. LCC_R1 and LCC_R2 are two replicates for LaGrue under control conditions. LCT_R1 and LCT_R2 are two replicates for LaGrue under HNT conditions.
